# Engineering of Advanced Materials for High Magnetic Field Sensing: A Review

**DOI:** 10.3390/s23062939

**Published:** 2023-03-08

**Authors:** Nerija Žurauskienė

**Affiliations:** 1 Department of Functional Materials and Electronics, Center for Physical Sciences and Technology, Sauletekio Ave. 3, 10257 Vilnius, Lithuania; nerija.zurauskiene@ftmc.lt; 2 Faculty of Electronics, Vilnius Gediminas Technical University, 10223 Vilnius, Lithuania

**Keywords:** colossal magnetoresistance, linear magnetoresistance, extraordinary magnetoresistance, low-field magnetoresistance, high-field magnetoresistance, manganites, 2D materials, graphene, silver chalcogenides, narrow band gap semiconductors, transition metal dichalcogenides, high-pulsed magnetic fields, magnetic field sensors, megagauss sensors, magnetoresistive sensors

## Abstract

Advanced scientific and industrial equipment requires magnetic field sensors with decreased dimensions while keeping high sensitivity in a wide range of magnetic fields and temperatures. However, there is a lack of commercial sensors for measurements of high magnetic fields, from ∼1 T up to megagauss. Therefore, the search for advanced materials and the engineering of nanostructures exhibiting extraordinary properties or new phenomena for high magnetic field sensing applications is of great importance. The main focus of this review is the investigation of thin films, nanostructures and two-dimensional (2D) materials exhibiting non-saturating magnetoresistance up to high magnetic fields. Results of the review showed how tuning of the nanostructure and chemical composition of thin polycrystalline ferromagnetic oxide films (manganites) can result in a remarkable colossal magnetoresistance up to megagauss. Moreover, by introducing some structural disorder in different classes of materials, such as non-stoichiometric silver chalcogenides, narrow band gap semiconductors, and 2D materials such as graphene and transition metal dichalcogenides, the possibility to increase the linear magnetoresistive response range up to very strong magnetic fields (50 T and more) and over a large range of temperatures was demonstrated. Approaches for the tailoring of the magnetoresistive properties of these materials and nanostructures for high magnetic field sensor applications were discussed and future perspectives were outlined.

## 1. Introduction

Commercialized magnetic field sensors are becoming some of the most important components in consumer, automotive, industrial, and other applications [1,2]. Particularly, the market share of magnetoresistive (*MR*) sensors is growing, as shown by the report from the Yole Développement on magnetic sensors market forecast from 2016 to 2022 [3] which predicted an increase of *MR* sensors market from 27% to 33% at the expense of widely used Hall sensors. The growth of *MR* technologies, named xMR (anisotropic AMR, giant GMR and tunnelling TMR) as well as giant magnetoimpedance (GMI) [4,5] is very fast due to the high sensitivity of these sensors, and reinforces the increase of their complexity and compatibility with semiconductor-based technologies [6]. However, magnetic sensors based on colossal magnetoresistance effect (CMR) are still at laboratory-scale development [7,8], despite the efforts to commercialize the CMR technologies [9]. Nevertheless, some CMR-based magnetic field sensors have already been developed up to 8th technological readiness level (TRL) for specific scientific applications in high-field measurements [10,11].

Moreover, the rapid growth of advanced technologies for the formation of thin films and nanostructures has resulted in increased research on the Lorentz force-induced magnetoresistance in various non-magnetic materials (metals, semimetals and semiconductors) and their nanostructures. Usually, the magnetoresistance values in non-magnetic materials are found to be low but suitable for certain applications only at cryogenic temperatures and high magnetic fields. At room temperatures (~300 K) and moderate magnetic fields (0.1–1 T) the *MR* determined as Δρ/ρ_0_ is usually negligible [12].

**Figure 1:**
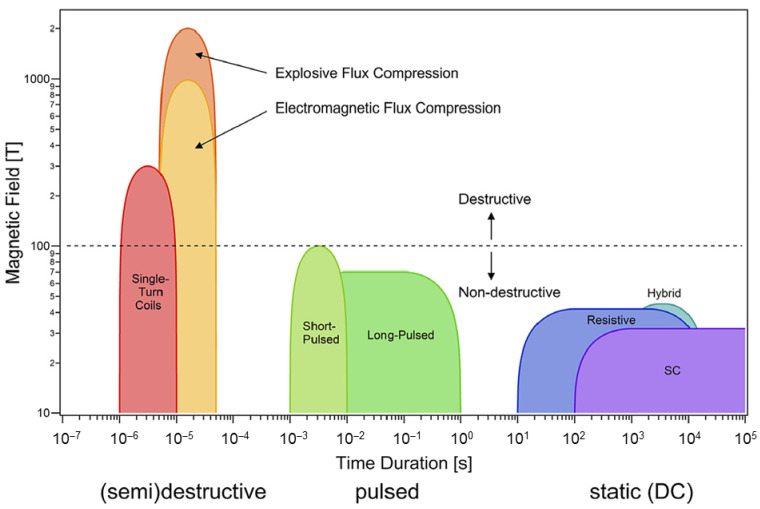
Overview of high magnetic field generation methods depending on amplitude and duration. (SC stands for Superconducting). Reprinted with permission from [13]. © 2018 Elsevier B.V.

It is important to note that each type of area of application has specific requirements for the sensor’s dimensions, device specifications such as detectivity, sensitivity, etc. and ranges of operation (magnetic field and temperature). Previous reviews on the magnetic sensors mainly focus on the technology of their structures, novel materials, and areas of applications for sensing of significantly low fields [1,4,5], and there is still a lack of overviews and outlooks on the magnetic sensors used for measurements of high magnetic fields. Figure 1 presents an overview of magnetic field ranges generated by advanced magnetic field generation techniques [13]. These methods are divided into static (DC), pulsed non-destructive and (semi)destructive. Advanced pulsed magnetic field generation technologies extend the magnetic field range for scientific, industrial, and medical applications [14,15]. Pulsed fields are usually generated with relatively short durations (microseconds—milliseconds) which are not always applicable in industry. The longer magnetic field pulses with high amplitudes can open new fields of applications. Recently, Matsui et al. [14] reported on the development of a new design for a mobile and low-cost pulse power supply used for generation of pulsed magnetic fields with amplitudes up to 24 T and pulse durations of ∼1 s. To measure such long-duration fields, sensors with good temperature stability are required. On the other hand, Kohama et al. [15] pointed out the need for advanced techniques for time-resolved measurements in high-pulsed magnetic fields to study the physical properties of materials, i.e., thermal conductivity, specific heat, critical current in superconductors, ultrasound, nuclear magnetic resonance, etc. The experimental accuracy of the measurable quantities depends also on the accuracy of measured magnetic field values during the entire pulse of the magnetic field. Therefore, high temporal resolution (to obtain a sufficient number of data points during the pulse) and measurement accuracy of magnetic field magnitude become very important parameters for magnetic sensing techniques used in high-pulsed magnetic field systems [11].

For moderate and high magnetic fields, pick-up coils are widely used [14,16,17]. Specially designed B-dots [18] or Faraday-effect magnetic field sensors [19] are applied in more specific equipment. However, to decrease the dimensions of the sensor and the measurement setup, solid-state based materials such as Hall sensors are preferable [20,21]. One can find more detailed information about Hall sensors in other review papers [22,23]. Nevertheless, the Hall effect configuration requires at least four electrodes, which is undesirable for pulsed magnetic field sensing. During the magnetic pulse, a large d*B*/d*t* induces large pick-up noise in the wires of the sensor, and it becomes difficult to extract the relevant data during a single measurement [21]. From this point of view, the magnetoresistive materials, whose structures can be designed with only two electrodes, are preferable [10,11]. Therefore, the choice of materials and engineering of nanostructures as well as the development of various sensor technologies which can be quickly adopted from the laboratory scale to commercial production are of great importance.

This review mainly focused on the investigations of thin films, nanostructures and two-dimensional (2D) materials exhibiting large magnetoresistance up to high magnetic fields (from ∼1 T up to megagauss). It will be demonstrated how the tuning of the nanostructure and the chemical composition of thin polycrystalline ferromagnetic oxide films (such as manganites) can result in a remarkable colossal magnetoresistance up to megagauss. Such films are used for the development of small-dimension high-pulsed magnetic field sensors capable of measuring the fields locally and independently of the magnetic field direction. Also, the influence of macroscopic disorder on the linear non-saturating magnetoresistive response over a large range of magnetic fields was demonstrated in two-dimensional (2D) Dirac semimetals, such as graphene, inhomogeneous conductors, such as non-stoichiometric silver chalcogenides, narrow bandgap semiconductors with very low effective mass and some other materials. The possibilities of tailoring the magnetoresistive properties of different solid-state materials and nanostructures for high magnetic field sensor applications at various ranges of temperatures were discussed and future perspectives were outlined.

## 2. Main Geometric Configurations of High-Field Magnetic Sensors Based on Solid-State Materials

**Figure 2:**
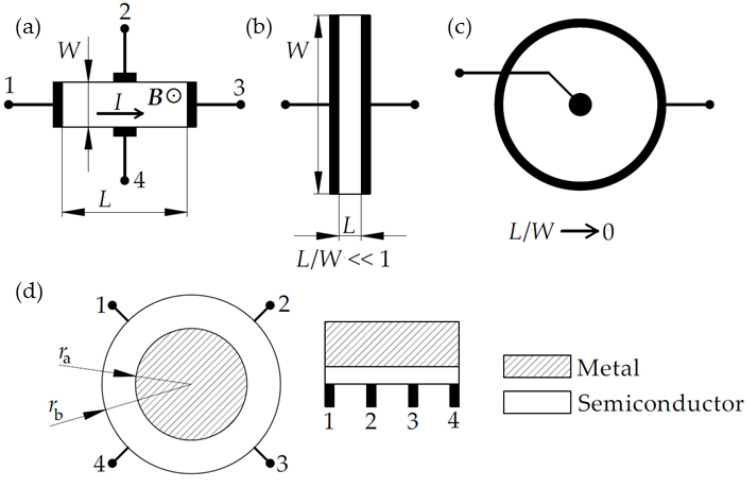
Main simplified configurations used for high-field magnetic sensors: (**a**) Hall-effect; (**b**) magnetoresistance; (**c**) Corbino disc; and (**d**) extraordinary magnetoresistance: van der Pauw disc geometry (**left**), bar shunt geometry (**right**).

In this Section, the main geometric configurations (see Figure 2) used for the fabrication of magnetic field sensors and the measurements of their main characteristics are discussed.

### 2.1. Hall Sensors

The Hall-effect sensors are the most-used magnetic field sensors due to their high sensitivity, linearity of response characteristics, operation in a wide range of magnetic fields, low cost, fabrication versatility, possibility of integration with CMOS technologies and other advantages [22]. The operation of Hall sensors is based on the Hall effect, which is a result of the Lorentz force ***F_L_*** appearing in a magnetic field ***B*** and deflecting the charge carriers of the flowing current in a solid conductor (or semiconductor) from the current direction: ***F_L_*** = q(***E*** + ***v*** × ***B***), where q and ***v*** are the charge of the carrier and its drift velocity vector, respectively, and ***E*** is the electric field vector. When the magnetic field is applied perpendicular to the surface of the conductor, the ***F_L_*** causes the deflection of the charge carriers and the accumulation of opposite sign charges at two opposite edges of the conductor, resulting in a voltage appearing perpendicular to the current flow. This voltage is called Hall voltage *V*_H_ and can be expressed as *V*_H_ = *R*_H_*IB*/*d*, where *I* is the current, *R*_H_ is the Hall coefficient, *p* is a concentration of charge carriers, *d*—thickness of the conductor (semiconductor). In the simplest case, when only one type of charge carrier is present and carrier scattering mechanisms are not taken into consideration, *R*_H_ = 1/(*qp*); in the case of n-type and p-type semiconductor, the *R*_H_ is more complex (one can find more details in review papers [22,24]).

It is well known that typical Hall-effect structures have Hall-bar geometry with a variety of modifications [24,25]. The simplified structure of rectangular parallelepiped geometry, as shown in Figure 2a, is not recommended, because the contacts (1 and 3 for current leads, 2 and 4 for Hall voltage measurement) must be soldered directly to the sample. Therefore, the Hall-bar or Hall bridge with extended arms is usually used. The Hall voltage is measured in-plane perpendicular to current direction when magnetic field ***B*** is applied perpendicular to the Hall-bar plane.

Recent technological advantages allow for the fabrication of micro-nanoscale Hall sensors which can be used for local magnetic field measurements in high-resolution magnetic imaging [23] and other applications. The main limitations of the Hall sensors in comparison to magnetoresistive sensors are related to the applications in the ultralow magnetic field range: the key-parameters—sensitivity and detectivity (the smallest magnetic signal which can be detected by a sensor)—are reported to be better for the magnetoresistive sensors [4]. Moreover, the power consumption of two-terminal *MR* sensors is lower in comparison to Hall sensors using at least four terminals. However, for high-field applications, in which detectivity and sensitivity is not as important, the Hall sensors with linear response characteristics are widely used, especially for DC measurements. Nevertheless, for high-pulsed fields, the four-terminal devices are not suitable due to high parasitic signals induced in the wires and cables of the sensors due to large d*B*/d*t* values. In such cases the two-terminal devices, based on magnetoresistance measurement, are preferable.

### 2.2. Magnetoresistive Sensors

#### 2.2.1. Conventional Magnetoresistance Configuration

For magnetoresistance measurements, the Hall voltage must be minimized. In this case, the sample with electrodes has to be short and wide, i.e., the distance between electrodes *L* has to be much smaller in comparison with the width of the sample *W*: *L*/*W* << 1 (see Figure 2b). This condition is satisfied for Corbino disc geometry (Figure 2c) which gives the highest physical magnetoresistance values because the Hall electric field is shorted, and *V*_H_ = 0. However, the Corbino disc geometry is inconvenient for device fabrication. Instead, the short and wide rectangular-shaped structures with *L*/*W* < 0.4 [24,26] could be used. For example, the Field-effect transistor (FET) configuration satisfies the requirement *L*/*W* << 1 well and is generally used in the magnetoresistance and *MR* mobility measurements (see, for example [27]).

The magnetoresistance is usually defined as

*MR* = 100% × [*ρ*(*B*) − *ρ*(0)]/*ρ*(0) (1)

where *ρ*(*B*) and *ρ*(0) are the resistivity at magnetic field *B* and zero-field resistivity, respectively. As the resistivity of the semiconductor increases in applied magnetic field, the *MR* is positive and can exceed hundreds and even several thousands of percent.

The origin of the magnetoresistance in ferromagnetic materials and nanostructures is different in comparison to semiconductors. More detailed information about AMR, GMR and TMR can be found in a review paper [4]. In this case, the resistivity of the structure decreases with the increase of the magnetic field due to the dependence of the spin-polarized current on the magnetization direction in magnetic layers, and the *MR* is negative. The aforementioned effects are employed for the development of highly sensitive magnetoresistive sensors operating in magnetic fields less than a few millitesla. Much higher magnetic fields can be measured by using Colossal magnetoresistive (CMR) materials in which the magnitude of negative magnetoresistance can be close to 100% (see Equation 1). It has to be noted, that for comparison of the large positive *MR* of semiconductors and negative *MR* (which is less than 100%) of ferromagnetic materials, one has to use relative resistance change, *ρ*(*B*)/*ρ*(0), instead of the *MR* (%). The origin of the CMR and main properties of these materials will be discussed in the Section 3.

The *MR* measurement of ferromagnetic materials is usually performed using four-terminal electrodes to avoid the influence of contact resistance. However, in pulsed fields the two-terminal configuration is preferable. Special technological conditions allow for the fabrication of samples with contact resistances of electrodes ~10^4^ times smaller in comparison to film resistance [28]. For the design of CMR sensors’ structures, see Section 3.2.

#### 2.2.2. Extraordinary Magnetoresistance Configuration

Extensive studies of the magnetoresistance effects and efforts to increase the *MR* values for sensor applications have resulted in the fabrication of semiconductor–metal hybrid structures exhibiting the so-called extraordinary magnetoresistance (EMR), which is a geometrical effect. The intrinsic magnetoresistance depends on the material properties (such as magnetization, mobility, electronic band structure), while the geometric contribution comes from the design of the device and the geometry of the electrodes used. The EMR was first reported by Solin et al. [29] in a four-terminal device fabricated from InSb disk of radius *r*_b_ and a concentric metallic shunt of radius *r*_a_, which short-circuits a current flowing through the middle metallic part of the device, when the magnetic field is zero (see schematic representation of van der Pauw disc geometry in Figure 2d, left drawing, current leads 1–2, voltage 3–4). In the applied magnetic field perpendicular to InSb plane, the charge carriers are deflected around the shunt and travel through the more resistive semiconductor material, resulting in enhanced magnetoresistance (by several orders of magnitude). The key parameter is the so-called filling factor *α* = *r*_a_/*r*_b_ whose values can be calculated to obtain the largest *MR* values in the structures. From a technological point of view, the disk geometry is inconvenient, especially for nanoscale devices. Therefore, another configuration—bar geometry—was suggested [30]. Its schematic drawing is presented in Figure 2d (right) which can be used with symmetrically or asymmetrically positioned current (*I*) leads and voltage (*V*) probes in different sequences: contacts labeled by 1,2,3,4 can represent *IVVI* or *IVIV* configurations [30,31,32]. Note that the reduced two-contact EMR device is also possible for nanoscale sensors applications [33]. In the bar-geometry, the length/width ratio of the semiconductor bar was found to be a critical geometric parameter to obtain the highest EMR values (see [30,32]).

The main focus of this review will be on the different classes of materials exhibiting magnetoresistance up to megagauss. As different scientific groups have reported magnetoresistance measurement results on various designs and configurations of the investigated structures and devices, one must be careful and take into account the used designs, when comparing the magnetoresistance values of different materials.

## 3. Colossal Magnetoresistance Materials

### 3.1. Lanthanum Manganites

Mixed-valence manganites with the perovskite structure (general formula

**Figure 3:**
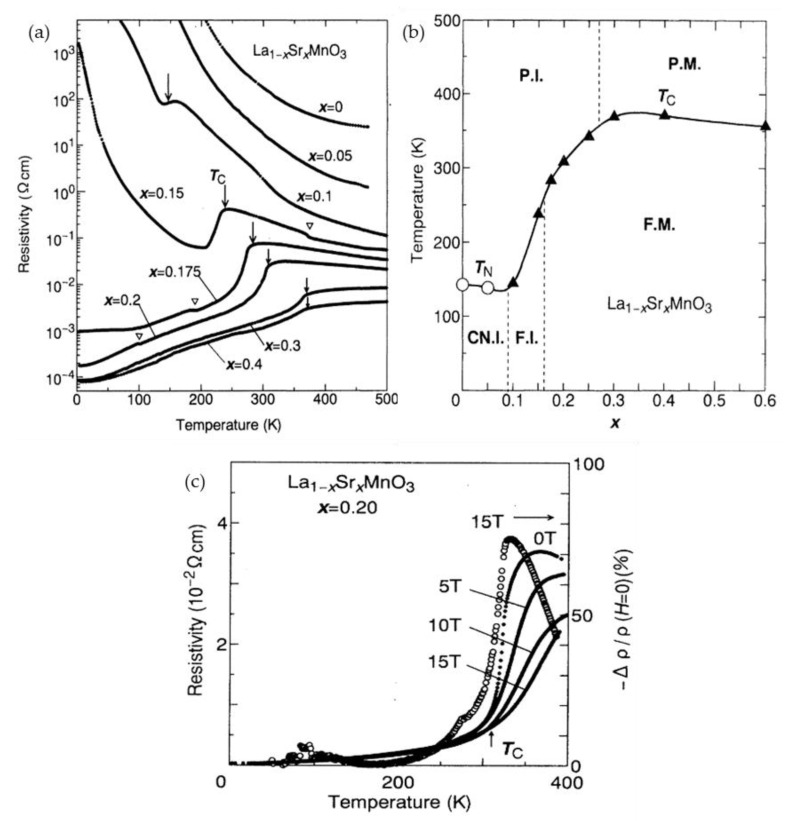
Main characteristics for La_0.8_Sr_0.2_MnO_3_ crystals: (**a**) resistivity vs. temperature dependences. Arrows indicate the critical temperature for the paramagnetic-ferromagnetic phase transition. Open triangles indicate structural transition; (**b**) electronic phase diagram. Open circles and filled triangles indicate the Neel (*T*_N_) and Curie (*T*_C_) temperatures, respectively. The meaning of abbreviations: paramagnetic insulator (PI), paramagnetic metal (PM), spin-canted insulator (CNI), ferromagnetic insulator (FI), and ferromagnetic metal (FM); and (**c**) resistivity vs. temperature dependence for chemical composition *x* = 0.20 under various magnetic fields. Reprinted with permission from [41], ©1995 American Physical Society.

R_1−x_A_x_MnO_3_,

where R rare-earth cation, A alkali or alkaline earth cation) have been widely studied starting from the investigations of La_1−x_Ca_x_MnO_3_ materials in the late 1940s performed at Philips research laboratories by Jonker and van Santen [34,35,36]. In these publications, the preparation of polycrystalline ceramic samples as well as investigations of their structural, electrical conductivity and magnetic properties were presented and discussed. A negative magnetoresistance in another lanthanum manganite—La_0.8_Sr_0.2_MnO_3_ obtaining the highest values close to the Curie temperature, was first presented and discussed by Volger [37]. The observation of the so-called “colossal” magnetoresistance (CMR) effect in the 1990s [38,39] on thin high-quality epitaxial films of doped manganites has again stimulated investigations of their properties. It was found that the CMR at room temperatures can be much larger (~60% at 5 T, normalizing the resistivity change to the zero-field value) [39] in comparison with multilayer structures of ferromagnetic/nonmagnetic materials [40], which were proposed for use in magnetic recording. The magnetoresistance (*MR*) is usually defined by Equation 1, therefore, for manganites it is negative, because the resistivity decreases with applied magnetic field. Figure 3 presents typical characteristics of one of the mostly investigated lanthanum manganite La_1−x_Sr_x_MnO_3_ crystals with different chemical composition (0 ≤ *x ≤* 0.6) [41]. Resistivity vs. temperature dependences reveal the insulator–metal transition (Figure 3a) which is not observed for *x* ≤ 0.05. The ferromagnetic–paramagnetic phase transition temperature (Curie temperature, *T*_C_, indicated by arrows) almost coincides with the metal–insulator transition temperature, *T*_M-I_, corresponding to a maximal resistivity *ρ*_m_ in *ρ* vs. *T* dependence. An electronic phase diagram is presented in Figure 3b showing different possible phases of this material. Figure 3c presents resistivity vs. temperature dependences at different applied magnetic fields and magnetoresistance at 15 T. One can see that the highest *MR* values are obtained in the phase transition region close to *T*_C_.

**Figure 4:**
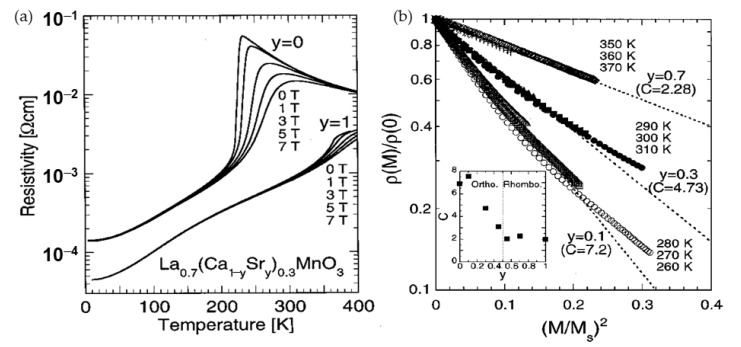
(**a**) Resistivity vs. temperature dependences for La_0.7_(Ca_1−y_Sr_y_)_0.3_MnO_3_ crystals with different chemical compositions. The observed *MR* at *y* = 0 is much stronger in comparison to *y* = 1; and (**b**) normalized resistivity represented on logarithmic scale as a function of the square of reduced magnetization for *y* = 0.1, 0.3, and 0.7. The inset shows the variation of a slope of this plot (constant C) with change of *y*. Reprinted with permission from [42], ©1995 American Physical Society.

The increase in magnetoresistance in manganites is usually found at the expense of the decrease of the *T*_C_ and *T*_I−M_. Figure 4a shows the temperature dependences of resistivity in La_0.7_(Ca_1−y_Sr_y_)_0.3_MnO_3_ crystals depending on Sr content *y* [42]. It is evident that for Ca-doped manganites (*y* = 0), in which *T*_M-I_ is shifted to lower temperatures, the magnetoresistance effect is much stronger. It should be noted that there is a correlation between the temperature-dependent resistivity changes and the magnetization of the material (*M*). The quadratic dependence on the reduced magnetization (*M*/*M*_s_) (*M*_s_ is saturation magnetization) was proposed for a ferromagnetic semiconductor taking into account thermal activation-type law [43]. The following empirical formula was proposed: *ρ*(*M*)/*ρ*(0) = exp[−C(*M*/*M*_s_)^2^], where C is a constant [41,42]. Figure 4b presents normalized resistivity dependence on the square of reduced magnetization for La_0.7_(Ca_1−y_Sr_y_)_0.3_MnO_3_ crystals [42]. The dashed lines indicate that the *ρ*(*M*)/*ρ*(0) could be approximated by this empirical relation for small values of the reduced magnetization. The scaling constant C was found in the range of ∼(2 ÷ 7) and depend on manganite doping.

The colossal magnetoresistance effect in manganites can be qualitatively explained by a double-exchange mechanism between manganese ions Mn^4+^ and Mn^3+^, as an applied magnetic field aligns their spins and, therefore, this leads to a decrease in resistivity. However, the double exchange model alone is not sufficient to explain the CMR effect, and in addition a strong electron-phonon interaction arising from the Jahn–Teller effect must be considered [44].

**Figure 5:**
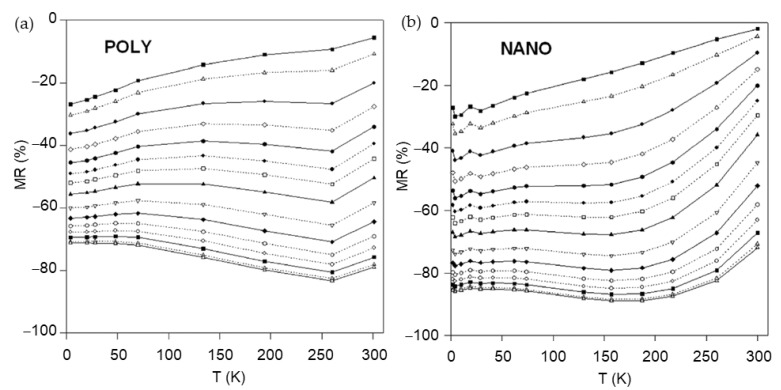
Temperature dependences of magnetoresistance for poly- (**a**); and nanocrystalline (**b**) manganites La_0.8_Sr_0.2_MnO_3_ upon applied different magnetic fields: 1, 2, 4, 6, 8, 10, 12, 15, 20, 25, 30, 35, 40, 45, and 47 T (from top to bottom). Reprinted with permission from [54]. Copyright 2015 AIP Publishing.

As the mixed-valence manganite perovskites exhibit very rich variety of crystallographic, electronic, and magnetic phases, the numerous investigations of the CMR effect in a variety of chemical compositions were performed. One can find more information in a comprehensive review by Coey [45] followed by other reviews with different aspects on preparation methods, chemical composition, structure and microstructure, material properties and various areas of applications [7,46,47,48,49,50,51,52,53]. It is worth mentioning the difference between the intrinsic and extrinsic properties of the manganites. Whereas intrinsic effects are found in the bulk single crystals or high-quality epitaxial films and are determined by material parameters, the extrinsic effects are only found in polycrystalline materials with defected structures having grain boundaries and artificial heterostructures [48]. Moreover, the high-quality crystals or epitaxial films exhibit the highest *MR* values only close to *T*_C_, which is a drawback for applications. Therefore, much attention was paid to polycrystalline or nanostructured films exhibiting so-called extrinsic magnetotransport phenomena [48]. The *MR* of such films is significant in a wide range of temperatures starting from *T*_M-I_ down to cryogenic temperatures (see Figure 5) [54].

It was found that the conducting mechanism in such materials is controlled not only by a double-exchange mechanism Mn^3+^–O^2−^—Mn^4+^, but also by the grain boundary resistivity and its relative quantity [55,56]. Polycrystalline bulk ceramics or thin films can be considered as a net of high crystalline quality grains (crystallites) and grain boundaries (GBs). As it is clear that the resistivity of polycrystalline materials is mostly determined by the properties of grain boundaries, many models were proposed to explain the magnetoresistance of GBs. Most of them were based on spin-polarized tunneling [57] of charge carriers through an insulating tunnel barrier or spin-dependent scattering of polarized charge carriers like in GMR structures. On the other hand, Evetts et al. [58] pointed out models based on “activated carrier transport”, such as variable range hopping (VRH), which is considered as resistance dependence on temperature for *T* > *T*_C_. In their review Evetts et al. [58] proposed to consider the GB as a mesoscale region with depressed *T*_C_ and saturation magnetization *M*_S_ and introduced a mesoscale magnetoresistive (MMR) response when magnetic field is applied. This approach could be valid for thicker GBs in comparison with a tunnel barrier of only a few nm thickness.

**Figure 6:**
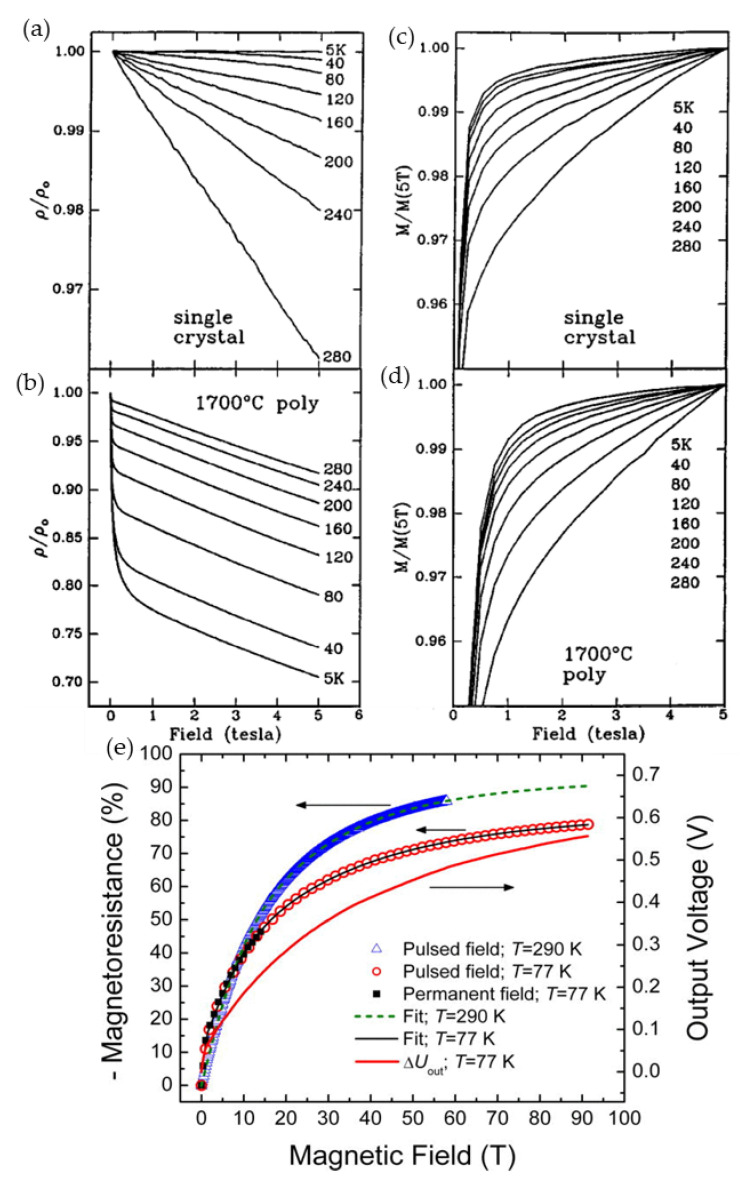
The magnetic field dependences of the normalized resistance (**a**,**b**) and magnetization (normalized to the 5 T value) (**c**,**d**); for La_2/3_Sr_1/3_MnO_3_ ceramic samples at various temperatures from 5 to 280 K. Reprinted with permission [57], ©1996 American Physical Society; and (**e**) *MR* vs. *B* dependences of nanostructured 400 nm thickness La_0.83_Sr_0.17_MnO_3_ film. Measurements performed in a static magnetic field (square symbols) and pulsed field (circular and triangular symbols corresponding to 77 K and 290 K temperatures, respectively). The solid red line (right scale) represents magnetic field dependence of output voltage (sensor’s response) when pulsed magnetic field of 91.4 T was applied. “Fit” curves show fitting results by using modified Mott’s hopping model taking into account contributions of nanocrystallites and grain boundaries. Reprinted with permission from [62], copyright 2012 IOP Publishing.

Most of the studies performed so far considered two main magnetic field ranges and related effects induced by a disorder in ferromagnetic perovskites: a low-field magnetoresistance (LFMR) and a high-field magnetoresistance (HFMR) [57,58]. Figure 6a presents the *MR* dependence of single crystal manganite without clear evidence of LFMR region, while Figure 6b shows an abrupt resistance decrease in low fields which is attributed to the LFMR effect and is mostly explained by spin-polarized intergrain tunneling. The HFMR could be recognized by almost linear variation of the resistivity with the field at higher fields with much lower slopes (see Figure 6b). It is attributed to the transport properties of disordered GB materials with reduced magnetization and *T*_C_ values [59] and thermal hopping of the charger carriers through the energy barrier as is predicted in the modified Mott’s variable range hopping (VRH) model [60,61,62].

For high-magnetic field-sensor applications, the polycrystalline manganite films are preferable, because their high-field magnetoresistance can be almost linear up to 7–10 T and non-linear, but not fully saturated up to megagauss [62] (see Figure 6c). Figure 6c presents magnetoresistance dependences on magnetic field at liquid nitrogen (77 K) and at room temperatures (290 K) performed at Dresden High magnetic field laboratory (HLD), Helmholtz-Zentrum Dresden-Rossendorf, Germany. The low-temperature *MR* was measured during 2011-year World record of 91.4 T set for nondestructive pulsed magnets [62]. It should be noted that for sensors applications in the megagauss field, the sensitivity at 91 T was sufficient: 2 mV/T (see response signal on the right scale). Room-temperature *MR* was measured up to 60 T and approximated by using modified Mott’s VRH model [61].

#### 3.1.1. Low-Field Magnetoresistance

As it was mentioned before, the observation of the low-field magnetoresistance at low temperatures is one of the main features of polycrystalline manganites [57]. In comparison with Giant magnetoresistance multilayered structures, the LFMR effect can extend the sensing magnetic field region up to hundreds of millitesla, what is an advantage for low-field magnetic sensors applications. Considering magnetic sensors for high-field measurements, the LFMR is important for pulsed field sensing, when not only amplitude, but also the whole pulse shape must be recorded. Lee et al. [58] predicted that the upper limit of conductivity change due to the LFMR effect could be up to 33.3% not depending on chemical composition. Therefore, during the last decades the major focus of scientific investigations has been on increasing the LFMR by introducing various artificial grain boundaries in thin-film structures to ensure a thin-energy barrier for spin-polarized tunneling. In some cases, an insulating thin layer was used (for example, SrTiO_3_ multijunction La-Sr-Mn-O/SrTiO_3_/La-Sr-Mn-O [63]), or composite manganite films were grown with a second insulating oxide phase of ZnO, NiO or CeO [64,65,66]. It was demonstrated, that for layered structure of LSMO/SrTiO3/LSMO the LFMR = 50% could be found at 4.2 K in magnetic field of 20 mT [63].

**Figure 7:**
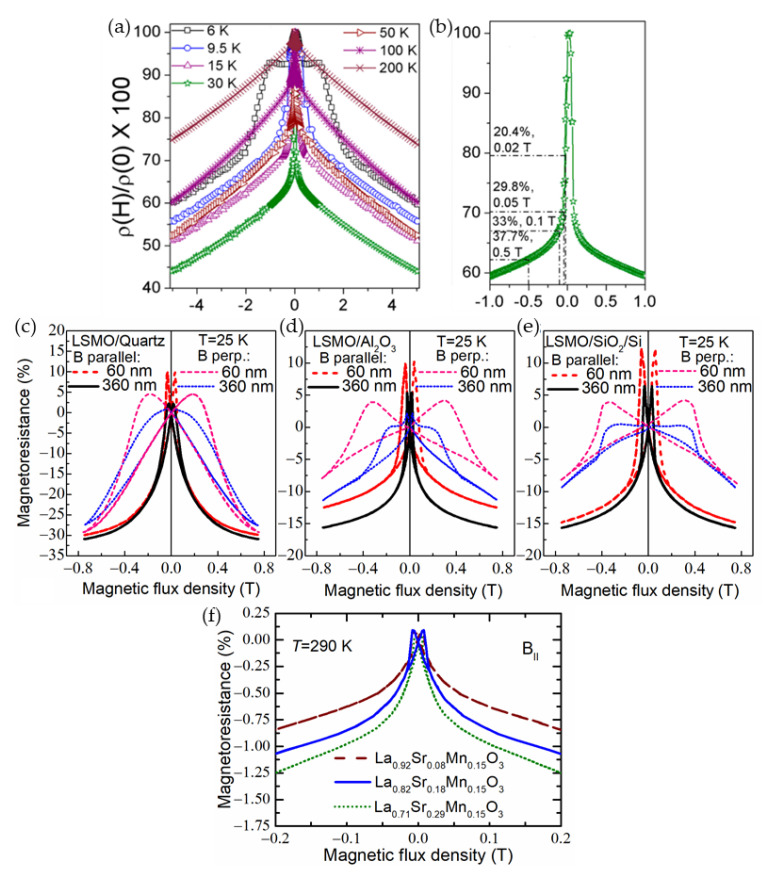
Normalized resistance as a function of applied field at different temperatures (**a**); and low-field magnetoresistance at 30 K (**b**) for La_0.71_Sr_0.29_MnO_3_ nanoparticles synthesized by nonaqueous sol–gel route. Reprinted with permission from [67], copyright 2014 American Chemical Society; (**c**–**e**) Low-field magnetoresistance dependences on magnetic flux density for different thickness films (60 nm and 360 nm) grown on different substrates. Measurements performed at ambient temperature of 25 K. Reprinted from [68], copyright 2022 MDPI; and (**f**) *MR* vs. magnetic field dependences at room temperature for nanostructured La_1−x_Sr_x_Mn_y_O_3_ films with different Sr content *x* and constant Mn excess *y* = 1.15. Reprinted from [69], copyright 2022 MDPI.

Although the LFMR effect is highest at low temperatures, the proposed methods increased the LFMR values also at room temperatures and led to the possibility of developing magnetic sensors operating at room temperatures. In [67], the authors presented their results on synthesized nanoparticles exhibiting enhanced LFMR = 30% at 100 mT (see Figure 7a,b) in comparison with large crystallites and bulk material. The comparison of LFMR values obtained from the literature of various magnetic nanostructures could be found in [67]. It is worth mentioning, that the adjustment of the film growth conditions and choice of substrate is very important for film quality and crystal structure and significantly influences the LFMR values. It was demonstrated that for single-layer polycrystalline La_0.83_Sr_0.17_Mn_1.21_O_3_ films grown on quartz substrate, the LFMR at 25 K showed twice higher values (31% at 0.7 T, see Figure 7c) in comparison to films grown on polycrystalline Al_2_O_3_ or SiO_2_ substrates (~15%) (see Figure 7d,e, respectively) [68]. Recently, it was demonstrated that Mn excess (*y* > 1) in polycrystalline La_1−x_Sr_x_Mn_y_O_3_ (LSMO) films increases the Curie temperature what results in LFMR effect at close to room temperatures [69]. It was found that the magnetoresistance of (–1.23 ÷ –0.8)% at temperatures (250 ÷ 290) K could be achieved at a magnetic field of 50 mT for La_0.7_Sr_0.3_Mn_1.15_O_3_ films without the introduction of an additional insulating phase, only by the adjustment of the chemical composition and film growth conditions (see Figure 7f).

#### 3.1.2. High-Field Magnetoresistance

**Figure 8:**
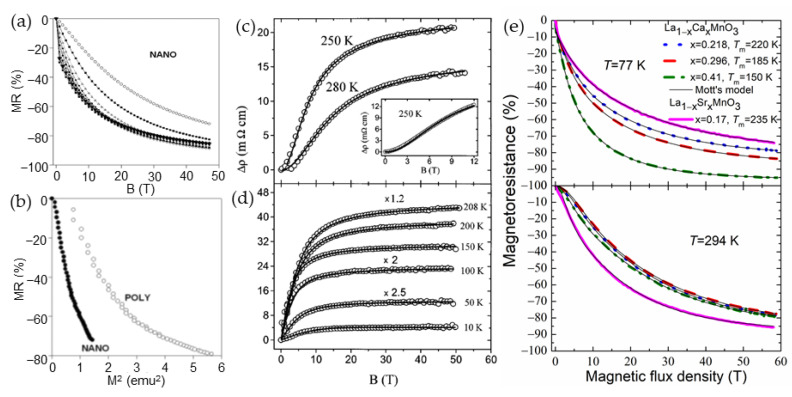
(**a**) Magnetic field dependences of magnetoresistance for nanocrystalline La_0.8_Sr_0.2_MnO_3_ manganites at temperatures 300, 260, 217, 187, 157, 130, 74, 62, 41, 29, 19, 10, 6.4, 4.2, and 1.7 K (from top to bottom); (**b**) the magnetoresistance as a function of a square of magnetization at 300 K for the poly- and nanocrystalline La_0.8_Sr_0.2_MnO_3_ manganites. Reprinted with permission from [54]. Copyright 2015 AIP Publishing; (**c**) magnetic field dependence measured in the pulsed field of the resistivity decrease in the paramagnetic (PM) phase. A thin solid line shows fitting by the square of the Brillouin function. The inset presents measurements in a superconducting magnet; (**d**) the resistivity decrease in the magnetic field fitted by the Brillouin function in the ferromagnetic (FM) regime. Curves at several temperatures are multiplied with the indicated figures for better clarity. Reprinted with permission [61], ©1998 American Physical Society; and (**e**) *MR* vs. magnetic flux density dependences in the FM (77 K) and PM (294 K) phases of nanostructured La_0.83_Sr_0.17_MnO_3_ and La_1−x_Ca_x_Mn_y_O_3_ films having different composition *x*. Thin solid curves represent fitting results to the modified Mott’s VRH model taking into account contributions of crystallites and grain boundaries: upper panel—fitting by the Brillouin function using Equation (2) at 77 K, lower panel—fitting by square of the Brillouin function using Equation (3) at 294 K. Reprinted with permission from [75]. Copyright 2014 IEEE.

Earlier investigations demonstrated that the high-field magnetoresistance depends on the size of crystallites of polycrystalline samples. Balcells et al. [70] investigated ceramic La_2/3_Sr_1/3_MnO_3_ samples having different grain sizes (their diameter was from 10 µm down to 20 nm) and found that the HFMR increases with decrease of the diameter of the grains. *MR* = 32% and *MR* = 50% in the field of 5.5 T was obtained for samples with average diameter of 10 µm and 25 nm, respectively. The linear dependence of the HFMR up to 5.5 T was explained by the existence of a noncollinear surface layer which thickness increases when grain size was reduced. The values of the *MR* observed in a large number of polycrystalline ferromagnetic perovskites are found of 80% and more at very high fields up to 50 T and above with no clear evidence of full saturation (see Figure 6e and Figure 8a) [54,62,71,72]. It should be noted that the magnetoresistance in high-quality manganites has tendency of saturation at approximately 7–10 T [61] (see Figure 8c,d). Figure 8a presents the *MR* dependences on magnetic field at different temperatures for nanocrystalline (mean crystallite size ~25 nm) manganites [54]. The highest *MR* absolute values at 47 T were found depending on temperature in the range of 85–89% for nanocrystalline films, while for polycrystalline ones (mean crystallite size ~155 nm) investigated during the same study [54], the *MR* absolute values of 71–83% were obtained. The authors evaluated the *MR* sensitivity in polycrystalline manganite films of about 3–5%/T at low fields and 0.2–0.4%/T at 47 T. For the nanocrystalline films, the corresponding sensitivity was 2.5–5% T and 0.2–0.6%/T, respectively.

Not fully saturating *MR* in nano-poly-crystalline manganites could be explained by the need of high fields for alignment of magnetic moments in grain boundaries which, due to disorder, are in a non-ferromagnetic (paramagnetic or antiferromagnetic) state in a wide range of temperatures. The highest values of the HFMR are usually found in the vicinity of *T*_M-I_. As mentioned before, there were many efforts to find the analytical expression for dependence of magnetoresistance or magnetoconductance on magnetic field. Simple linear dependences of conductance at high fields were explained by Lee et al. [58] and expressed as follows: [*G*(*H*) − *G*(0)]/*G*(0) = (1/3) *M*^2^ + 2χ_GB_*HM*, where *G*(0)—zero-field conductance, *M*—normalized intragrain magnetization, χ_GB_ normalized susceptibility of the GB region, *H*—magnetic field strength. Indeed, linear magnetoconductance was measured in magnetic fields of up to 47 T [71], and at low temperatures ≤ 100 K the curves showed a clear linear behavior. It was found that the slope of the normalized conductance *G*(*H*)/*G*(0) decreases with increase of temperature. The dependence of magnetoresistance magnitude proportional to –*M*^2^ was found for a number of manganite materials. At lower magnetization region, this proportionality was valid for large grain polycrystalline as well as small-grain nanocrystalline manganites (see Figure 8b) [54]. For higher magnetization region, a change in dependence character could be observed (at *MR* values of about 50–60%, which could be explained by the domination of the second term in [*G*(*H*) − *G*(0)]/*G*(0) expression, which is linear on *M* and depends on the grain boundary susceptibility.

The observed decrease in resistivity of polycrystalline films is dominated by the grain boundary (GB) effects. The lack of saturation measured up to 50–60 T was explained by various models. It could be attributed to strong antiferromagnetic coupling of spins in the GBs. The high-field magnetization has been studied in Sm_0.5_Ca_0.5_MnO_3_ manganites and almost constant increase up to high magnetic fields up to 100 T was found [73]. These films exhibited metamagnetic transitions, which were attributed to the field-induced collapse of the charge-ordered state. It should be noted that such antiferromagnetic manganites cannot be used for very high magnetic field sensors, because the metamagnetic transitions is a drawback for calibration of such sensors.

The attempts to scale the conductance change as a linear function of magnetic field for polycrystalline films were not successful. Gangineni et al. [74] investigated conductance dependences for a number of bulk and thin film manganites of varied composition and found, that a quadratic polynomial dependence *G*(*H*) = a + b*H* + c*H*^2^ with temperature-dependent coefficients a, b, and c well describes *G*(*H*) up to 47 T. However, a deeper physical background was sought. Due to low carrier mobility of manganites, their conductivity is governed by Mott’s variable range hopping mechanism. Therefore, Wagner et al. [60,61] proposed to modify Mott’s VRH model by taking into account the dependence of hopping barrier on misorientation of hopping electrons at an initial and a final state, and demonstrated, that *MR* of epitaxial Nd_0.52_ Sr_0.48_ MnO_3_ films scales proportionally to the Brillouin function ℬ(*x*) in the ferromagnetic (FM) state and to ℬ^2^ (*x*) in the paramagnetic (PM) state in a wide range of magnetic field, where *x* = gμ_B_*J*(*T*)*B*/k_B_*T*, is the ratio of magnetic and thermal energy, g is the Lande factor, μ_B_ is the Bohr magneton, k_B_ the Boltzmann constant, *B* the magnetic flux density, *T* is the temperature, and *J*(*T*) is the average spin moment at the hopping sites. Figure 8c,d presents results of the measurements fitted by the proposed scaling up to 50 T. Later, Mandal et al. presented magnetoresistance results for La_2/3_Sr_1/3_MnO_3_ thin films measured up to 20 T over a wide range of temperatures. The authors obtained high value of spin moment *J* = 60 using the scaling the *MR* dependences by Brillouin function in the vicinity of the phase transition and attributed it to the presence of large magnetic clusters due to the short-range ferromagnetic ordering. Considering nanostructured films (polycrystalline with nano-size crystallites) having wide (7–10 nm) mesoscopic regions of grain boundaries, Balevicius et al. [62] proposed to use a simple approach, where both crystallites and grain boundaries are connected in series. In such case the magnetoresistance was analyzed by the sum of the two contributions:

*MR* = *f* × *A*_C_ × ℬ (*x*_C_) + (1 − *f*) × *A*_GB_ × ℬ (*x*_GB_) + LFMR for FM (2)

*MR* = *f* × *A*_C_ × ℬ^2^ (*x*_C_) + (1 − *f*) × *A*_GB_ × ℬ^2^ (*x*_GB_) for PM state (3)

where *f* is the crystallite-material fraction, (1 − *f*) is the grain boundary fraction. ℬ(*x*) is the Brillouin function, *x*_C(GB)_ = gμ_B_*J*_C(GB)_*B*/k_B_*T*. The spin-orbit quantum numbers of the crystallites (*J*_C_) and grain boundaries (*J*_GB_), and the magnetoresistance amplitudes (*A*_C_) as well as (*A*_GB_), respectively, are used as parameters. Low-field magnetoresistance (LFMR) in this study was obtained from the low-field measurements performed in magnetic fields aligned parallel to the film plane. The fitting results for La_0.83_Sr_0.17_MnO_3_ nanostructured film up to 91.4 T are presented in Figure 6e. This approach was used to analyze lanthanum manganite nanostructured films doped with different Ca amounts [75]. 

Figure 8e presents experimental and fitting results both for the ferromagnetic (77 K) and paramagnetic (294 K) states. The fitting parameters for the La-Ca-Mn-O films were obtained in the following ranges: *f* × *A*_C_ = −(31.3–45.4)%, (1 *– f*) × *A*_GB_ = −(44.9–45.7)%, *J*_C_ = 7.5–11.8, *J*_GB_= 1.8–3.1, *LFMR* = −(5–7)% at *T* = 77 K; *f* × *A*_C_ = −(28.8–30.7)%, (1 *– f*) × *A*_GB_ = −(52.9–57)%, *J*_C_ = 27.2–37.8, *J*_GB_ = 7.8–8.6 at *T* = 290 K. The obtained *J* values indicate that the crystallites and GB material in these films behave like a superparamagnet of magnetically aligned polarons, which is possible only for wider GBs.

It should be noted that many attempts to increase magnetoresistance values in various ranges of temperatures and magnetic fields were made by changing substrates, growth conditions, film thickness, chemical composition and doping elements at A-site and B-site (general formula ABO_3_) of polycristalline ferromagnetic perovskites.

**Figure 9:**
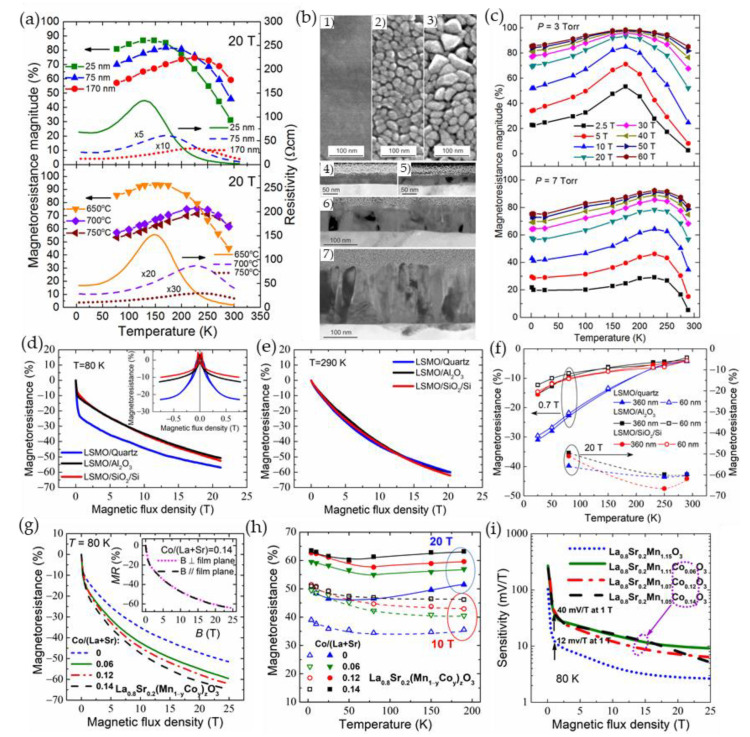
Some examples of magnetoresistance (or its magnitude) (*MR*), sensitivity (S) and resistivity (ρ) dependences on temperature (*T*) and/or magnetic flux density (*B*) for nanostructured manganite films: (**a**) La-Sr-Mn-O grown on glass ceramics: upper panel—different film thickness, lower panel—different film growth temperature; left scale—*MR*(*T*), right scale—ρ(*T*); (**b**) SEM surface images (1, 2, 3) and low-magnification cross-sectional bright-field TEM images (4, 5, 6, 7) of LSMO films having different thicknesses grown at 750 °C: 25 nm (1, 4, 5);  75 nm (2, 6); 170 nm (3, 7). Reprinted with permission from [56]. Copyright 2018 Springer; (**c**) La-Ca-Mn-O films prepared at different gas pressure in the MOCVD chamber conditions: upper panel—3 Torr, lower panel—7 Torr. Reprinted with permission from [76]. Copyright 2017 IEEE; (**d**,**e**) *MR*(*B*) up to 20 T (pulsed-field) for La-Sr-Mn-O films grown on different substrates. Inset in (**d**) presents low-field region. (**f**) *MR*(*T*) for low- and high-field regions. Reprinted from [68]. Copyright 2022 MDPI. (**g**–**i**) La-Sr-(MnCo)-O films with different Co amount; (**g**) *MR*(*B*) dependences, inset—different direction of magnetic field (parallel and perpendicular to film plane); (**h**) *MR*(*T*) dependences at 10 T and 20 T; and (**i**) sensitivity S(*B*)dependence when voltage supply of 2.5 V was used. Reprinted with permission from [77]. Copyright 2021 Elsevier.

Figure 9a presents the correlation of magnetoresistance vs. temperature dependences (left scale) with resistivity maximum of polycrystalline La_0.83_Sr_0.17_MnO_3_ films (right scale) grown by pulsed injection MOCVD on glass ceramics substrates [56]. One can see that at high magnetic fields the *MR* magnitude is weakly dependent on the temperature in a wide range down to metal–insulator transition temperature *T*_M-I_ and is highest at temperatures close to *T*_M-I_. Figure 9b presents SEM and TEM images of films having different thicknesses. It is evident from Figure 9a,b, that by changing the microstructure of the film it is possible to tune the resistivity and *MR* values in a wide range of temperatures. Similar MR(*T*) behavior is also observed in Figure 9c, in which the nanostructured La_1__−__x_Ca_x_Mn_y_O_3_ (LCMO) films prepared at different conditions were investigated [76]. In some cases, the increase of the *MR* in the low temperature range could be found, in which usually upturn in resistivity vs. temperature dependences is observed, especially for more disordered films (see, for example, Figure 5b). At low temperatures the high-field magnetoresistance values are also dependent on the LFMR effect which could have a significant influence as presented in Figure 9d (at high temperatures its influence disappears with decrease of LFMR, Figure 9e. Figure 9f summarizes LFMR and HFMR values obtained for nanostructured La_1__−__x_Ca_x_Mn_y_O_3_ films grown on different substrates. The most significant influence of the used substrate is observed at low temperatures and low fields, when grain boundary properties determine the spin-polarized tunneling peculiarities in the films. Figure 9f–h presents example, how magnetoresistance dependences on magnetic field, temperature and its sensitivity, respectively, depend on La-Sr-Mn-O film doping in so-called B-site (substitution of Mn by Co) [77,78]. One can see that higher amount of Co increases the *MR* values, what could be explained by partly destroyed double exchange hopping Mn^3+^−O^2−^−Mn^4+^, when cobalt ions substitute manganese in the lattice, which results in increased resistivity. It is worth noting that one must be careful when increasing the disorder in polycrystalline films—by doping with Co or other elements, or by using other means such as decreasing of grain size [79]. The increase of disorder by reducing the grain size could lead to collapse of the VRH and drive the system in the opposite direction of the Anderson localization. As a result, the suppressing the CO/AFM state and growth of the FM contribution would lead to the decrease of resistivity [79]. Such behavior could have a great influence on magnetoresistance values and sensors operation at low temperatures.

Table 1 presents typical magnetoresistance values in high magnetic fields at low and high temperatures. As numerous investigations were performed on manganites with various doping elements, Table 1 also presents some typical results reported on La_0.67_Ba_0.33_MnO_3_ [80], La_0.4_Gd_0.1_Ca_0.5_MnO_3_ [81], La_0.45_Ho_0.05_Ca_0.5_MnO_3_ [82], La_0.45_Ca_0.55_MnO_3_ [83] and La_0.5_Ca_0.4_Li_0.1_MnO_3_ [84] materials not discussed more detailed in this review.

### 3.2. High-Field Magnetic Sensors Based on CMR Effect

Widely used AMR, GMR or TMR sensors are limited in magnetic field sensing range [1,4,85] and cannot be used to measure moderate or high-amplitude fields. Each application for magnetic field measurement requires a set of specific parameters, such as magnetic field and temperature ranges of operation, sensitivity, measurement accuracy, dependence on magnetic field direction, frequency of operation etc. For high-field measurement, the detectivity is not so important.

**Figure 10:**
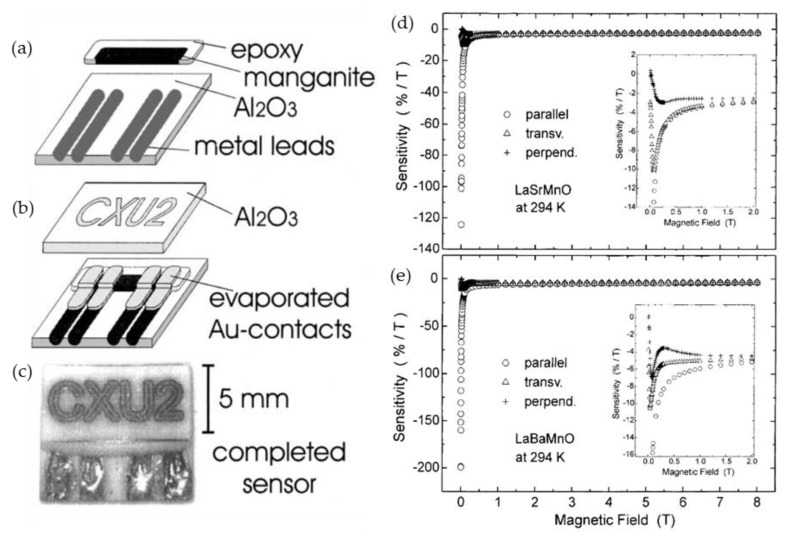
(**a**–**c**) Schematic drawing of LaBaMnO bulk sensor; (**d**,**e**) field sensitivity dependence on magnetic field applied in different directions (parallel, transverse and perpendicular) for LaSrMnO (upper panel) and LaBaMnO films (lower panel). Reprinted with permission from [80], copyright 2001 Elsevier.

Xu et al. [80,86] demonstrated, that although the low-field magnetoresistance values are high in polycrystalline manganite films, it is highly anisotropic, when magnetic field is longitudinal, transverse or perpendicular in respect to current direction and film plane. However, at higher fields (>2−30 T) [80], the *MR* exhibits an isotropic behavior. The authors reported on magnetic field sensor (see Figure 10a), for which the similar behavior is found for field sensitivity (see Figure 10b) defined as *MR* change with magnetic field: *S*_MR_ = 100% ×(1/*R*_0_)(Δ*R*/Δ*B*), where 100% × (1/*R*_0_)(Δ*R*) is magnetoresistance of the film. One can see that at room temperature the maximal sensitivity of ~200%/T is achieved for La-Ba-Mn-O films in the range of 1–3 mT. At a high magnetic field the absolute value of *S*_MR_ is decreased to 5%/T. The authors concluded that nearly isotropic behavior is very promising for sensors applications in high magnetic fields. From this point of view, the CMR sensors are preferable for some applications in comparison with Hall sensors which sense only one component of the field, especially when the field direction is not known in advance and probing areas are very small (impossible to use three sensors installed in different directions).

**Figure 11:**
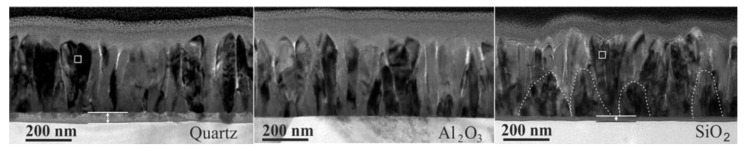
TEM images of nanostructured La-Sr-Mn-O films grown on quartz, polycrystalline Al_2_O_3_ and amorphous SiO_2_/Si substrates. Reprinted from [68], copyright 2022 MDPI.

It is important to note, that with technological achievements and possibilities of nanoscale structural modification, the tunning of magnetoresistance anisotropy became possible. In [87] the authors demonstrated very high enhancement of in-plane magnetic anisotropy in La_0.67_Sr_0.33_MnO_3_ films when the top layer of the film was patterned into nanoscale periodic stripes. From the other hand, to diminish the anisotropy, the growth of nanostructured manganite films with column-shaped nano-crystallites perpendicular to film plane was proposed [56]. One can find the examples of TEM images in Figure 9b and Figure 11.

**Figure 12:**
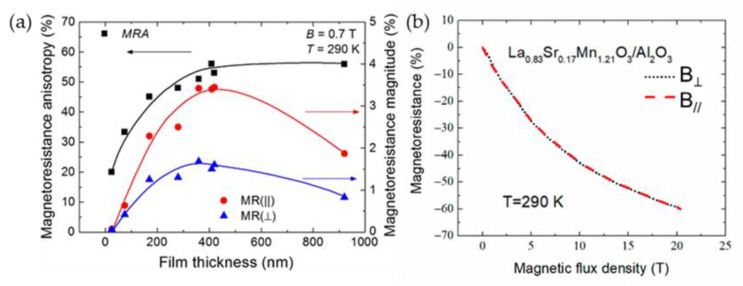
(**a**) The dependences magnetoresistance anisotropy MRA (left scale) and magnetoresistance *MR* in perpendicular (⊥) and parallel (||) to film plane field directions (right scale) on thickness of La_0.17_Sr_0.83_MnO_3_ films grown on glass ceramics substrate. Reprinted with permission from [56]. Copyright 2018 Springer; and (**b**) magnetoresistance dependences on magnetic flux density for La_0.17_Sr_0.83_Mn_1.21_O_3_ film grown on polycrystalline Al_2_O_3_, when the magnetic field was applied parallel and perpendicular to film plane. Reprinted from [68]. Copyright 2022 MDPI.

For thin polycrystalline films, the main reason for anisotropy is related to the demagnetization field (due to geometry factor) [88]. In the case of column-shaped thin films, the total magnetization direction of the film is in-plane, while the magnetization direction of a single crystallite is along its vertical axis and directed perpendicular to the film plane. Therefore, these directions can partly compensate each other (if in-plane long-range interaction between crystallites is somehow reduced by disordered grain boundaries). The tuning of magnetoresistance anisotropy is possible by changing film substrates [68] or thickness [56] which determines dimensions of crystallites (width of crystallite columns) (see Figure 11). Figure 12a presents magnetoresistance anisotropy MRA dependence on film thickness of La_0.17_Sr_0.83_MnO_3_ films grown on glass ceramics substrate. In addition, the dependences of the magnetoresistance in perpendicular and parallel directions are also presented. The MRA is defined as follows [56]: MRA *=* 100% × (MR(||) − MR(⊥))/MR(||), where MR(||) and MR(⊥) are magnetoresistance absolute values when magnetic field is oriented parallel and perpendicular to the film plane, respectively. One can see that for thinnest films which magnetoresistance in both directions is negligible, the MRA is smaller; however, these films could not be used for sensors applications due to very small MR. At high fields, the MRA is negligible (see Figure 12b) and this isotropic property was applied for the development of so-called CMR-B-scalar sensors based on nanostructured manganite films [10].

**Figure 13:**
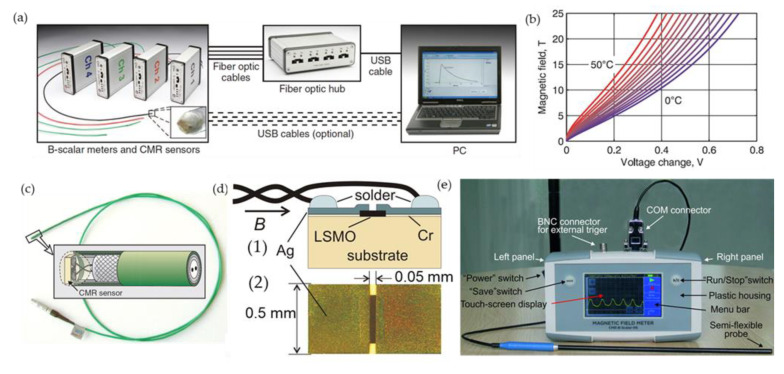
(**a**) The outside view and schematic diagram of four-channel pulsed magnetic field measurement system based on CMR-B-scalar sensors. Optic fiber cables are used for transfer recorded and stored in the channel signal to PC. (**b**) Family of calibration curves for a particular CMR-B-scalar sensor. (**c**) Schematic 3D cross section of the CMR-B-scalar sensor with cable (not to scale). Reprinted with permission from [10]. Copyright 2014 AIP Publishing. (**d**) Schematic drawing of the cross section of two-terminal sensor with bifilarly twisted wires (1) and its picture with two Ag electrodes before soldering wires (2). Reprinted with permission from [62], copyright 2012 IOP Publishing. (**e**) Outside view of a hand-held CMR-B-scalar-06 magnetometer with a color touch screen for a waveform display. The hand-held semiflexible probe is connected to the device. Reprinted with permission from [98]. Copyright 2020 IEEE.

Figure 13a presents high pulsed magnetic field measurement system [10]. It consists of 4 sensors connected to 4 measurement modules (digital channels). The recorded signal is stored in the module and could be transferred to a personal computer. The calibration curves of one of the probes are presented in Figure 13b. Two electrodes with bifilarly twisted wires (Figure 13c,d) ensure minimized parasitic signal due to the induced electromotive force proportional to the magnetic field derivative. This system can be used for magnetic field distribution measurements using an array of CMR-B-scalar sensors positioned at a certain distance each from the other. The signal is synchronized and recorded simultaneously by recording channels. The magnetic field measurement system was used for measurements of magnetic field distribution and diffusion during operation of electromagnetic launchers [18,89,90], highly inhomogeneous transient fields in a coil gun [91], field distribution in nondestructive pulsed magnets [92], welding quality control in magnetic forming and welding systems [93]. Despite the fact that the resistance of manganite film, which is a basic element of the sensor, is dependent on ambient temperature, the CMR-B-scalar sensors can be successfully calibrated in advance in a wide range of temperatures (see Figure 13b) and the data are stored in the software of the system what allows simultaneous conversion of the measured signal (voltage) to magnetic field values [10]. It should be noted that the operation temperature range of the sensors could be increased by using nanostructured manganite La_1__−__x_Sr_x_Mn_y_O_3_ films with Mn excess (*y* > 1) [11,94]. For films with *y* = 1.15, the largest *MR* values were obtained at *T* = 363 K in a magnetic field of 20 T [11,94]. As mentioned before, Mn excess in manganite films increases the Curie temperature, which results also in increased low-field magnetoresistance at close to room temperatures. For the stability of the probes, a special ageing procedure is performed, which ensures long-term stability of resistance and magnetoresistance of manganite films used for sensor fabrication [95,96,97].

Another modification of magnetic field meter is presented in Figure 13d [98]. 

It could be used for continuous DC, AC and pulsed magnetic field measurements. This probe consists of two sensors, magnetic and temperature, and the latter is used to take into account temperature variations during the measurement. The design of this probe ensures measurement of the temperature in the vicinity of the magnetic field sensor. The calibration data in this case are stored in the EEPROM chip of the probe. This device can be used not only in laboratory, but also industrial conditions.

## 4. Linear Magnetoresistance in Silver Chalcogenides and Narrow Bandgap Semiconductors

### 4.1. Magnetoresistance in Nonstoichiometric Silver Chalcogenides

The growing demand of magnetic sensors for measurement of higher magnetic fields in scientific, industrial, or medical equipment resulted in a search of materials exhibiting high magnetoresistance values in a wider magnetic field range in comparison to magnetoresistive AMR, GMR and TMR sensors. The capabilities of CMR-based sensors were demonstrated in the previous section. It was demonstrated that despite the magnetoresistance tendency to saturate in high magnetic fields, the CMR-sensors can be successfully used up to megagauss [62]. However, the increase of linear magnetoresistance range would be very useful for sensor applications. Some history on the observed linear magnetoresistance in non-magnetic materials and theoretical models for its explanation can be found in [99]. In this Subsection, the properties of a class of magnetoresistive compounds of non-magnetic materials, namely, silver chalcogenides, will be described.

High-quality stoichiometric Ag_2_S, Ag_2_Se or Ag_2_Te show no appreciable magnetoresistance, however, adding a very small amounts of excess silver (approximately one part in 100,000), the magnetoresistance of nonstoichiometric silver chalcogenides increases linearly with applied field (linear magnetoresistance, LMR) without saturation up to very high fields (55–60 T) [100,101,102,103,104]. For the explanation of the LMR, Abrikosov [99,105] proposed a model which assumes that the material is inhomogeneous with clusters of excess Ag atoms and could be considered as a gapless semiconductor with a linear energy spectrum. Such type magnetoresistance was called “quantum magnetoresistance”. Quantum effects could be observed at low temperatures in high magnetic fields, when so-called “extreme quantum limit” [99,105] is achieved. In such case the cyclotron energy has to be larger than the Fermi energy *E*_F_: ħω_c_ > *E*_F_, where ħ is reduced Plank’s constant, ω_c_ is cyclotron frequency. Therefore, all electrons are supposed to coalesce into the lowest Landau level which results in the direct proportionality of *MR* and *B*.

Parish and Littlewood (PL model) [106,107] developed a classical model in which some defects or impurities could cause mobility fluctuations and the LMR is caused by macroscopic inhomogeneities. They demonstrated that geometric effects of the inhomogeneities could be responsible for the nonsaturating magnetoresistance. A model was based on a two-dimensional random-resistor network which was capable of simulating a macroscopic level. In [108] the authors expanded the 2D random-resistor network to three dimensions thus representing current distortions in the thickness dimension. The experimental data on Ag_2 + δ_Se and Ag_2 + δ_Te were well explained by using this model [108].

**Figure 14:**
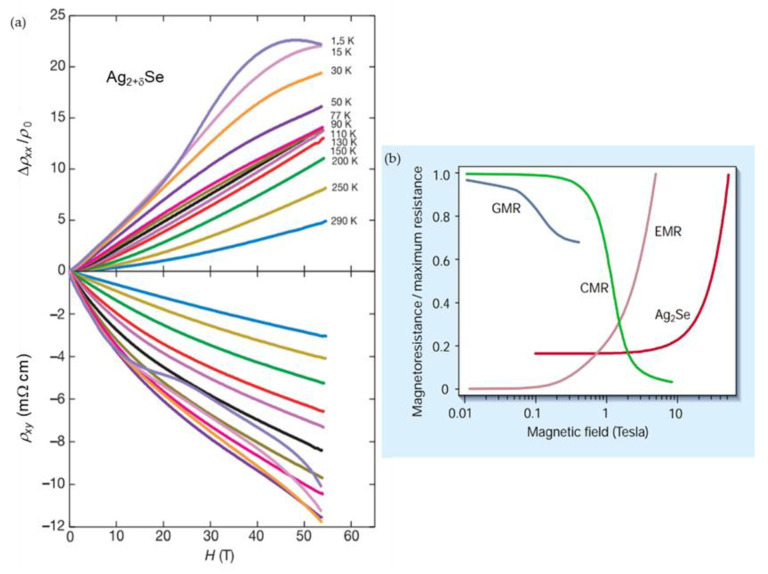
(**a**) Magneto-transport of Ag_2 + δ_Se with δ ~ 10^−24^ measured in a 55-T pulsed magnetic field. Upper panel: the magnetoresistance vs. magnetic field at different temperatures. Lower panel: field dependence of the Hall resistivity at the same temperatures (color coded). In the low-*T*, low-*H* limit, electron density *n* = 1.1 × 10^18^ cm^−3^. Reprinted with permission from [104]. Copyright 2002 Springer; and (**b**) performance of magnetic-field sensors. The dependence of magnetoresistance vs. applied magnetic field is shown for typical devices based on giant magnetoresistance (GMR) at a temperature of 295 K, colossal magnetoresistance (CMR) at 220 K and extraordinary magnetoresistance (EMR) at 300 K. Reprinted with permission from [109]. Copyright 2002 Springer.

As mentioned before, the technological progress in the development of high-pulsed nondestructive magnets has driven the search for high-field magnetic sensors. Husman et al. [104] proposed a “megagauss sensor” based on nonstoichiometric silver chalcogenides. The scientists issued a patent on the sensor’s design (No. US 6,316,131 B1) and published a paper on the LMR investigations [104]. Figure 14a presents the *MR* measured up to 55 T in a wide temperature range. Large *MR* values at room temperature show that silver chalcogenides can be competitive with the CMR materials. Soh et al. [109] compared performance of GMR, CMR, silver chalcogenide (Ag_2_Se) and EMR magnetic field sensors and presented a dependence of magnetoresistance normalized to maximum resistance on applied magnetic field (see Figure 14b). It was shown that magnetic sensors based on nonstoichiometric silver chalcogenides may provide better performance in a high magnetic field range in comparison to GMR or CMR sensors.

After the demonstration of unique properties of nonstoichiometric Ag_2_Se, Thomas F. Rosenbaum 20 years ago declared “the quest for imperfection” [110] and discussed how tiny imperfections on microscales could unusually affect the properties of materials on macroscales and their responses to different external stimuli (magnetic field in this case) leading to the design of scalable devices. Since then, the investigations of other groups of materials with some introduced “disorder” were extensively carried out.

### 4.2. Magnetoresistance in InSb

The observed linear magnetoresistance in different class of materials could be converted to a linear function which allows easy calibration of magnetic field sensors. Narrow-gap semiconductors is another class of materials exhibiting the LMR. A typical representative is InSb [111], in which the unusual electronic properties result from low carrier concentration, small electron effective mass and long mean free path of charge carriers. As discussed by Hu and Rosenbaum [111], in InSb the realization of “extreme quantum limit” is possible due to existence of “tiny pockets of the Fermi surface with a small effective mass”. The authors proposed to use only a lightly doped single crystal of InSb with only one dominating carrier type. In such cases the “extreme quantum limit” can be reached at 2 T. The authors demonstrated how tunning of impurity doping level enables to control and achieve quantum linear magnetoresistance in a wide range of temperatures (50−175 K) and magnetic fields (0.7−13 T) reaching (20,000−40,000) % at 13 T.

**Figure 15:**
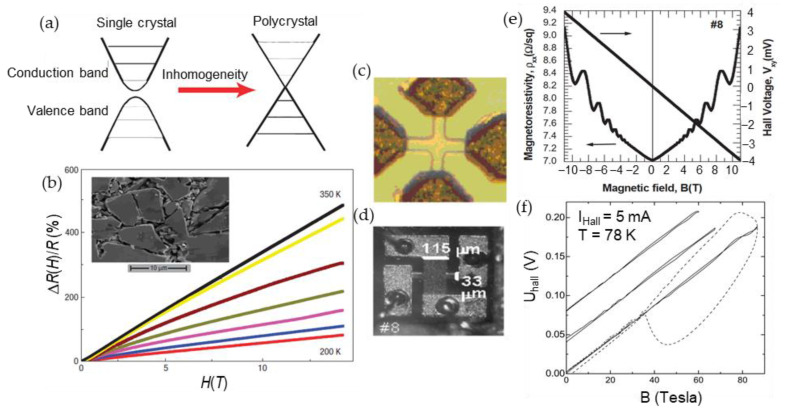
(**a**,**b**) Linear classical magnetoresistance in macroscopically inhomogeneous InSb: (**a**) inhomogeneities create tails in both the conduction and valence bands and cause them to overlap; (**b**) the linear magnetoresistance in the temperature range of (200–350) K. Inset: SEM image of an InSb polycrystal with typical grain size of 10–20 µm. Reprinted with permission from [111]. Copyright 2008 Springer; (**c**) picture of micro-Hall probe (MHP) based on Sn-doped n-InSb/i-GaAs MBE-grown heterostructures. The chip dimensions: 0.5 × 0.5 mm^2^ with square sensitive area 20 × 20 µm; (**d**) the mesa of the MHP chip (0.6 × 0.6 mm^2^ and sensitive area 115 × 33 µm). Reprinted with permission from [21]. Copyright 2010 Springer; (**e**) magnetoresistivity (left scale) and Hall voltage (right scale) for MHP measured at *T* = 1.1 K ($I_{AC}^{13\;Hz}$ = 50 μA). The SdH oscillations in the Hall data are invisible due to the scale of this graph. Reprinted with permission from [112]. Copyright 2004 Elsevier; and (**f**) Hall signals measured in pulsed magnetic field. Solid lines: corrected Hall signal at three pulses (shifted). Dashed line: as-measured signal at the 87.2 T pulse. Reprinted with permission from [21]. Copyright 2010 Springer.

At and above room temperatures, the magnetoresistance should be diminished due to increased phonon scattering. However, this limitation can be overcome by introducing some disorder in the material (classical PL model [106,107]). In this case, the fluctuation of mobility, rather than mobility itself, will determine the *MR*(*B*) behavior. Figure 15a presents a schematic drawing of the conduction and valence bands of a single crystal and polycrystalline InSb, indicating that the inhomogeneities cause their overlapping. Figure 15b shows linear classical magnetoresistance of polycrystalline InSb with introduced droplets of Sb as macroscopic inhomogeneities [111]. The *MR* of ~500% was found at 350 K at 14 T opening the possibilities for high-temperature high-field magnetoresistive devices.

As it was mentioned before, the geometric manipulation of the sample structures could increase the LMR [29,113]. Branford et al. [113] demonstrated this possibility in InSb epitaxial layers grown on GaAs substrate. They investigated magnetotransport properties of InSb structures fabricated in Corbino, EMR and disk geometries and observed the increasing linearity of the magnetoresistance with increasing number of disc arrays, what is predicted by classical PL model. It was found that the EMR geometry produces the highest *MR* values at low field, while the Corbino geometry allows to achieve the largest non-saturating linear magnetoresistance values at high fields.

Another configuration—microminiature Hall sensors—was proposed by Mironov et al. [21,112] for pulsed magnetic field measurements up to megagauss (see Figure 15c,d. Figure 15e presents magnetoresistance and Hall voltage dependences on magnetic field up to 11 T for microminiature Hall sensors based on Sn-doped n-InSb/i-GaAs MBE-grown heterostructures [112]. One can see, that at a low temperature (1.1 K) the quantum oscillations are superimposed on the *MR* dependence, while they are not observed in Hall voltage dependence due to the presented field range. It is worth mentioning that for sensors applications at cryogenic temperatures, the quantum oscillations can be suppressed by a special nanostructure design [114]. Vasil’evskii et al. [114] demonstrated that by introducing higher concentration of structure defects in nano-sized heterostructures (InSb/i-GaAs, InAs/i-GaAs) the Shubnikov–de Haas oscillations (SdHO) at cryogenic temperatures (1.5 ÷ 4.2) K could be diminished and linear response of Hall signal is obtained up to 14 T, while the single-crystal whiskers of the same material (InSb, InAs) show distinct SdH oscillations. The authors concluded that the heterostructure-based sensors could be used in modern particle accelerators and fusion reactors at strong magnetic fields (*B* > 3 T) conditions.

It was found that Hall sensors show small temperature variations over wide range of temperatures (1.1–300) K and pulsed magnetic fields (up to 52 T). This property is very promising for the development of magnetic probes for cryogenic temperature applications. Figure 15f presents three measurements of pulsed magnetic fields up to 87 T [21]. One can see, that during single pulse, the high pick-up voltage induced in the wires of the probe due to high d*B*/d*t*, is superimposed on the signal (dashed curve). To extract useful signal, the measurement has to be performed twice pulsing with opposite polarities [21]. As it was demonstrated by Stankevic et al. [11], it is possible to get useful response signal of microsecond duration and avoid induced pick-up voltage by using a bipolar-pulse supply to drive the sensor and processing the data during the pulse.

**Figure 16:**
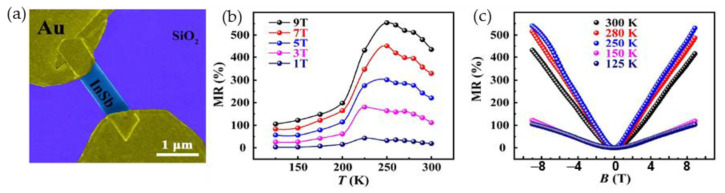
(**a**) SEM image (false-colored) of two-terminal InSb nanosheet; (**b**) temperature dependence of *MR* different magnetic fields; and (**c**) the *MR* dependences on magnetic field at different temperatures. Reprinted with permission from [105]. Copyright 2020 IOP Publishing.

To diminish parasitic pick-up voltage during measurements of high pulsed magnetic fields, a two-electrode configuration is preferable [11]. Tong et al. [115] proposed free-standing InSb single-crystal nanosheets with only two electrodes for future magnetic field sensors with nanoscale dimensions of the sensor’s sensing area. Figure 16a presents scanning electron microscopy (SEM) image of the proposed device. Figure 16b,c shows *MR* dependences on temperature and magnetic field, respectively, up to 9 T. One can see that such a linear MR, which is almost insensitive to temperatures in the range of 250–300 K might have potential practical applications in magnetic field sensors operating at and around room temperatures [115].

## 5. Single-Few-Layer Graphene

### 5.1. Magnetoresistance in Graphene

**Figure 17:**
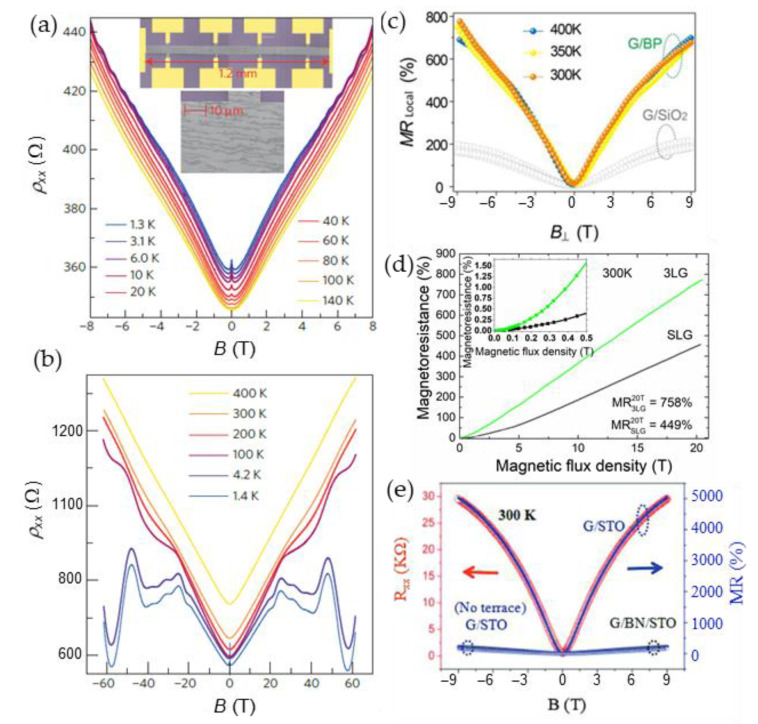
(**a**) Linear *MR* (resistivity) measured in large-area bilayer graphene. Inset presents the SEM image of a Hall bar together with a close-up that shows mainly bilayer, but also trilayer strips as darker shaded areas; (**b**) *MR* (resistivity) measured in pulsed magnetic fields up to 62 T at different temperatures (data have been vertically shifted by 20 for better visibility). Reprinted with permission from [128]. Copyright © 2015, Nature Publishing Group; (**c**) magnetoresistance (*MR*) of graphene/black phosphorus (G/BP) and G/SiO_2_ devices at various temperatures. Reprinted with permission from [123]. Copyright 2018 American Chemical Society; (**d**) the *MR* dependence on magnetic field (pulsed) up to 21 T of single- and three-layer graphene at 300 K. The inset: The *MR*(*B*) dependence up to 0.5 T for single- and three-layer graphene. Reprinted from [12]. Copyright © 2015, Nature Publishing Group; and (**e**) longitudinal resistance *R*_xx_ and *MR* as a function of a magnetic field near the charge neutrality point (CNP) for G/STO with terraces (red and blue lines), G/STO without terrace (blue dashed line), and G/BN/STO (black dashed line). Reprinted with permission from [124]. Copyright 2020 Wiley-VCH GmbH.

Future miniaturization of magnetic sensors leads to the search of advanced materials, in which magnetic field induced phenomena are significant when reducing dimensions to nanoscale. Although the magnetic sensors based on three-dimensional bulk materials or thin films exhibit relatively high magnetoresistance and sensitivity to magnetic fields, the dimension reduction to nanoscales is sometimes a challenge for device fabrication. The engineering of advanced materials on the nano-microscales also leads to new phenomena and increased functional possibilities of such materials [7]. During the last decades the attention to two-dimensional (2D) materials, such as graphene, which is a 2D Dirac semimetal and exhibits very high mobility of charge carriers [116], has rapidly increased. Graphene demonstrates remarkable electronic, magnetic, mechanical, and thermal properties and can be used for a number of different applications [117,118,119]. For magnetic sensing and magnetoelectronics, the large positive magnetoresistance induced by the Lorentz force has attracted attention of many scientific groups [120,121,122,123,124]. In low magnetic fields (*B* < 1 T), the *MR* has classical quadratic dependence on *B* [12,111,120] and normally must saturate to a relatively small magnitude. However, due to various inhomogeneities it can show non-saturating linear dependence in higher fields [120,121]. As it was overviewed in previous sections, the linear magnetoresistance (LMR) in high magnetic fields was observed in a number of different 2D and 3D materials, such as nonstoichiometric silver chalcogenides [108] and narrow bandgap semiconductors [111]. It was also found in a large number of different classes of two-dimensional materials such as, for example, 2D electron gas in GaAs quantum wells [125], magnetic topological semimetals as MnBi [126] or topological insulators as Bi_2_Te_3_ [127]. Different scientific groups reported on the LMR in few-layer or multilayer graphene even at room and above temperatures [12,120]. Kisslinger et al. [128] measured reproducible LMR in bilayer graphene up to 62 T with some peculiarities induced by quantum effects at low temperatures, which could obscure the linear dependence (see Figure 17a,b). Figure 17 presents some examples of the LMR observed in different graphene single or multilayer structures.

As it was mentioned in previous Section 4, different theories were proposed to explain the LMR in graphene. Hu and Rosenbaum [111] proposed that for a disordered conductor both classical [106,107] and quantum effects [105,109] could compete and both models could explain the observed effects under certain conditions. The quantum origin of the LMR could be proven by observation of Shubnikov–de Haas oscillations and quantum Hall effect which are superimposed on the LMR dependence on the magnetic field and are more pronounced at low temperatures in high magnetic fields, when so-called “extreme quantum limit” [99,105] is achieved. Friedman et al. [120] explained the observed results by quantum model. In classical approach (Parish and Littlewood model) some defects or impurities could cause spatial charge inhomogeneities, which introduce mobility fluctuations and increases magnetoresistance values proportionally to the applied magnetic field. In the PL model the crossover magnetic field *B*_c_, at which the transition from quadratic to LMR is observed, is proportional to inverse Hall mobility *µ* and should increase with temperature. In addition, the slope d*MR*(*T*)/d*B* ∝ *µ*(*T*).

It was shown that the LMR can be tuned by changing Hall mobility by various methods, for example, by adsorption or desorption of gas molecules in graphene by annealing it in helium atmosphere [122]. Usually at room temperatures in intermediate fields (~9 T) the magnetoresistance is observed in the range of 50–200% in single layer graphene prepared on SiO_2_ [123] or SiC [122] substrates. Many attempts were made to increase the magnetoresistance values by decorating monolayer graphene surface with ferromagnetic transition metals adatoms (Co) [129], choosing special substrate (black phosphorus [123] (see Figure 17c) or boron nitride [130]. Larger magnetoresistance could be more likely observed in few layer-by-layer stacked [12,131] (see, for example, Figure 17d) or multilayer [132] graphene because of increased level of its disorder and interlayer interactions. In the latter case, when few-layer graphene structure is used, the two/multiband model [132,133] has to be considered. According to it, when more than two conducting channels with different charge carrier mobility (*µ*) exist, the current at low magnetic field flows through the channel with higher mobility. When magnetic field is increased, the current will flow mostly through the channel with lower mobility, because the conductivity of the channels decreases as 1/[1 + (*µB*)^2^].

Gopinathan et al. [132] studied few-layer graphene/boron–nitride heterostructures and observed extremely large magnetoresistance, which was considered as local and non-local. The authors defined the local magnetoresistance as arising from differential carrier mobility across various layers of graphene upon a normal magnetic field. The non-local magnetoresistance was supposed to be due to the magnetic field induced Ettingshausen–Nernst effect [132]. Very high *MR* values (see Table 2) are promising for magnetic sensors applications. An interesting approach was proposed by Hu et al. [124] to increase the *MR* by laminating single layer graphene (G) on a terraced substrate, such as TiO_2_-terminated SrTiO_3_ (STO). The *MR* of 5000% was observed at 9 T at room temperatures (see Figure 17e).

Table 2 presents some examples of magnetoresistance in intermediate and high magnetic fields observed in single, few-layer or multilayer graphene prepared on different substrates.

One of the important parameters of 2D-material based magnetic sensors is the magnetoresistance anisotropy. As the charge carriers are confined in the 2D film plane, the action of a Lorentz force when applying magnetic field perpendicular to the film plane results in the maximal magnetoresistance value. In the case of in-plane magnetic field, it would be hard to change the trajectories of charge carriers by the action of Lorentz force in the direction of film thickness. An anisotropic magnetoresistance effect was reported in few-layer graphene stacks performing vertical transport measurements, when the angle *θ* between magnetic field and graphene plane was changed [134]. Taking into account both quadratic and liner *MR* (*B*) dependences, the experimental data of resistance dependence on *θ* at *B* = 14 T were well fitted by considering the vertical component of the field *B*_⊥_cos(*θ*), where *B*_⊥_ is magnetic field perpendicular to the graphene stack plane (*θ* = 0). The carrier mobility values calculated from the fitting results were related to the carrier transport through the graphene stack corresponding to combined effects of both perpendicular and in-plane charge movement. It was found that the higher the mobility is, the larger the *MR* magnitude could be obtained. Liu et al. have demonstrated [135] that the AMR in graphene can be optimized by tuning sample shape, electrode distribution and gasses adsorptions. It was demonstrated that a two-fold symmetric AMR dependence on the angle between magnetic field and graphene plane observed at high temperatures could be changed into a one-fold dependence at low temperature for a graphene sample with an irregular shape. This “anomalous” AMR behavior was explained by an anisotropic scattering of carriers from asymmetric edges and the boundaries of electrodes which could serve as active adsorption sites for gas molecules at low temperatures. The AMR effect observed in graphene could be used for the developing of ultra-thin AMR devices.

### 5.2. Magnetic Sensors Based on Graphene

**Figure 18:**
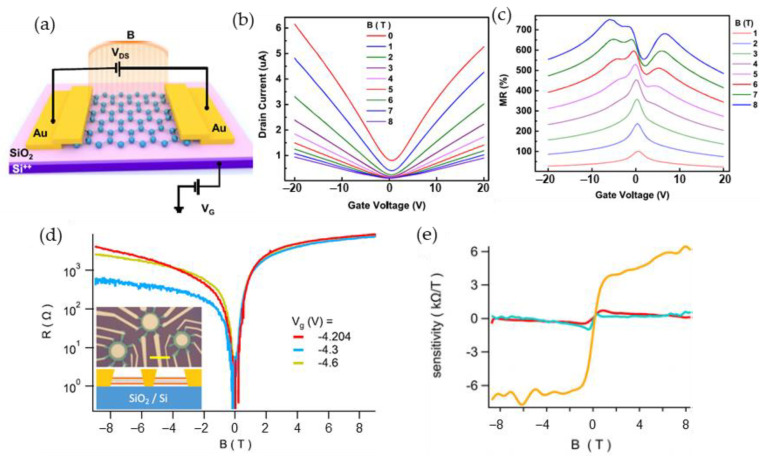
(**a**) A schematic drawing of a gated-sensor based on single-layer graphene in a two-probe configuration. *V*_G_ is gate voltage, *V*_DS_ is drain-source voltage, *B* is normal magnetic field; (**b**) drain current; (**c**) magnetoresistance dependences on *V*_G_ at different magnetic field values. Reprinted from [138]. Copyright © 2021, American Chemical Society; (**d**) resistance changes of EMR-device with magnetic field at different gate voltages Vg. The inset shows a microscopic image of three EMR-devices fabricated from a single graphene/hBN structure with contacts and central metallic shunt, and the schematic drawing in the side-view geometry; and (**e**) four-terminal sensitivity of devices keeping fixed outer radius of 2.9 µm but changing shunt-to-outer radius ratios: 0.38 (highest, yellow line), 0.63 (blue), 0.75 (red). Reprinted from [139]. Copyright © 2021, AIP Publishing.

Technological control of disorder in graphene resulting in high magnetoresistance values observed at room and above temperatures opens new possibilities for the development of graphene-based magnetic field sensors. For sensing permanent magnetic fields, usually Hall, Field effect transistor (FET) or Extraordinary magnetoresistance (EMR) configurations are used. Although these sensors have 3–4 electrodes, the use of a single-few layer graphene allows to miniaturize the sensors down to nanoscales [31,136,137]. Figure 18 presents some examples of such structures/devices and their main characteristics. For a gated sensor based on single-layer graphene (Figure 18a) the drain current is dependent on the magnetic field when the gate voltage is changed (Figure 18b) [138]. One can see that the *MR* values (Figure 18c) also depend on *B* and gate voltage. Figure 18d presents resistance change on *B* at different voltages, when the structure is designed in an EMR configuration [139]. 

As was discussed in previous sections, the EMR effect has attracted a lot of attention in recent years due to the significant increase of magnetoresistance due to the geometrical effect [29,113]. The EMR structures consist of a high-mobility material layer (usually semiconductor) and a nonmagnetic metal shunt. Using this shunt, the current path is changed upon the application of external magnetic field. This effect far exceeds the magnitudes of magnetoresistance of the materials using the usual configurations of electrodes. An inset in Figure 18d shows a device structure with van der Pauw electrodes and a concentric metal disk. One can see that the EMR value (Figure 18d) and sensitivity to the field (Figure 18e) are greatly dependent on the ratio of the radius of the metal disk and outer radius of the graphene film. Usually, before the fabrication of EMR-structures, simulations are performed to obtain the optimal ratio of the radii under different magnetic fields.

**Figure 19:**
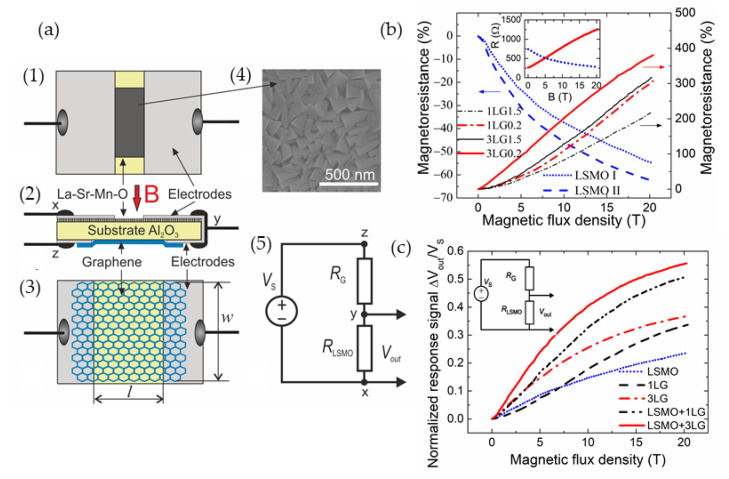
(**a**) (1)–(3) Schematic representation of manganite/graphene sensor. Magnetic field (*B*) applied perpendicular to the layers. (4) SEM image of LSMO film. (5) Electrical circuit of the sensor. *R*_G_ and *R*_LSMO_ are resistances of graphene and manganite, respectively. vs. = 1.25 V. (**b**) *MR* vs. *B* of manganite films deposited by pulsed injection MOCVD using one (LSMO I) or two (LSMO II) sources of precursors (left scale), and single- three-layer graphene (1LG or 3LG, respectively), when distance between electrodes was 1.5 mm (LG1.5) and 200 µm (LG0.2) keeping the same width/length ratio (w/l) = 3 measured at *T* = 293 K. The inset presents resistance vs. *B* change of LSMO II and 3LG. (**c**) Normalized voltage response vs. *B* for manganite/graphene sensor composed of LSMO II and 1LG or 3LG with dimensions 200 × 600 µm^2^. The voltage response from individual manganite (LSMO) and graphene (1LG and 3LG) elements are also presented. Reprinted from [140]. Copyright © 2021, AIP Publishing.

A different approach was suggested for increasing the magnetoresistance and corresponding response signal of a sensor by combining into one hybrid structure a single-few-layer graphene exhibiting positive magnetoresistance and lanthanum manganite film exhibiting negative magnetoresistance [12,140,141]. Figure 19a (1)–(3) drawings present such structure prepared on both sides of the same Al_2_O_3_ substrate, which ensures the very small sensing area of the magnetic sensor [140]. Figure 19a (4) presents the SEM image of the manganite LSMO film. In this study, graphene was transferred onto the substrate by using wet transfer. The electrodes marked by *x*, *y*, *z* ensure connection of the structure in a simple circuit, see Figure 19a (5), with two resistors (graphene *R*_G_ and manganite *R*_LSMO_). Upon application of external magnetic field, the *R*_G_ increases and *R*_LSMO_ decreases (see inset in Figure 19b) resulting in increased output voltage measured across the *R*_LSMO_ (see Figure 19c). One can compare the response signals of single elements (only manganite LSMO, single-layer graphene 1LG, three-layer graphene 3LG), connected with ballast resistor of constant resistance value, with the response signal of a combination of these films. The maximum response is obtained for hybrid LSMO + 3LG structure and it does not saturate up to 20 T. The highest sensitivity of 72 mV/VT, defined as *S* = d(Δ*V*_out_/*V*_S_)/d*B*, is obtained in the range of (1–3) T; however, it is significant in a wide range of magnetic fields (13 mV/VT at 20 T). Therefore, the design of this sensor, when both manganite and graphene layers are prepared on both sides of the same substrate, enabled to scale the useful area of the sensor down to 0.16 mm^3^. These dimensions can be minimized to micrometer scale by growing graphene directly on the substrate. However, the sensitivity in such cases is expected to decrease, because the electron mean free path in grown on substrate graphene is on the order of few micrometers (depending on the quality) [142,143]. However, for sensing with high spatial resolution, the sensitivity would be still significant [31]. Pisana et al. [136] demonstrated the sensitivity (normalized by current) in the range from −250 up to 100 V/AT depending on gate voltage of the graphene EMR sensor over ±0.5 T. Thus, the sensors with capabilities of nanoscale spatial resolution can be designed using graphene.

## 6. Two-Dimensional Transition Metal Dichalcogenides

**Figure 20:**
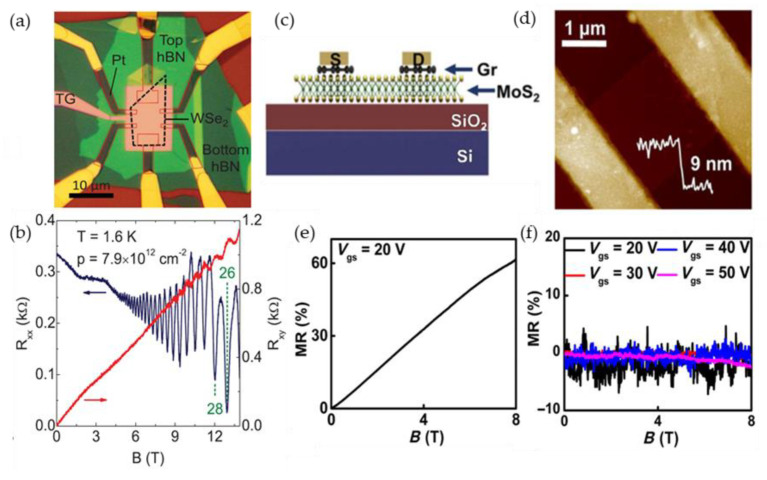
(**a**) Encapsulated monolayer WSe_2_ device; (**b**) Shubnikov–de Haas oscillations in the longitudinal (*R*_xx_) and Hall (*R*_xy_) resistances of WSe_2_ device measured as a function of the magnetic field at a temperature of 1.6 K. SdHO appear in *R*_xx_ starting at magnetic field of ~4.5 T. The steps appearing in *R*_xy_, indicate the quantum Hall states. Reprinted from [146]. Copyright © 2016, American Physical Society; (**c**) the cross-sectional view of the MoS_2_ FET; (**d**) the AFM image of the MoS_2_ FET; (**e**) the magnetoresistance dependence of MoS_2_ FET on magnetic field at constant gate voltage *V*_gs_ at 2K; and (**f**) the *MR* of pure MoS_2_ FET without graphene insertion. Reprinted from [147]. Copyright © 2020, Tsinghua University Press and Springer-Verlag GmbH Germany, part of Springer Nature.

With numerous investigations of graphene properties, there has been great interest in other graphene-like materials, such as 2D semiconducting transition metal dichalcogenides (TMDCs), which show great potential for applications in spintronics, optoelectronics and photonics [144,145]. The general chemical formula of TMDC is MX_2_ being M—transition metal element, such as Mo, W, and X—chalcogen element, such as S, Se and Te. Figure 20 presents some examples of magnetoresistive devices based on TMDC materials and measurements of resistance and magnetoresistance in applied magnetic field. One can see that for the encapsulated monolayer WSe_2_ device (Figure 20a) at low temperatures, quantum effects emerge in the linear longitudinal resistance dependence [146] (Figure 20b). Huang et al. [147] proposed a MoS_2_ field-effect transistor (FET) with graphene insertion layer (see Figure 20c,d). It should be noted that a number of studies on magnetoresistance and transport properties in TMDC materials is limited by moderate electrical performance of metal contacts in these devices. In [147], the graphene layer was used to ensure high-quality contact interface.

The obtained gate-tunable non-saturation linear *MR* was 67% in magnetic field of 8 T at 2 K (Figure 20e). The authors measured FET characteristics with insertion graphene layer and pure MoS_2_ FET (Figure 20f) and explained the linear *MR* by the effects of the contact interface between the graphene and MoS_2_ layers. For comparison, the *MR* of pure MoS_2_ FET without graphene insertion is presented in Figure 20f. Taking into account the proportionality of the magnetoresistance slope of the MoS_2_ FET with graphene layer to the magnitude of mobility, and crossover field to inverse mobility, the origin of the observed LMR was explained by the classical Parish–Littlewood model.

## 7. Summary and Outlook

In this review, the past and current investigations of the typical classes of magnetic and non-magnetic materials exhibiting large magnetoresistance values up to megagauss were overviewed and discussed.

As was pointed out in the roadmap of magnetoresistive sensors development [4], the *MR* sensor market is growing each year at the expense of Hall sensors in different application areas, especially when enhanced sensitivity, detectivity and miniaturization is required. The magnetoresistive sensors, as they are broadly understood, usually include xMR (AMR, GMR, TMR) and GMI devices, and their operation range of magnetic fields is less than tens of millitesla. In this review, the *MR* sensors were considered with a broader meaning—materials which exhibit large magnetoresistance values in a wide range of magnetic fields.

With the rapid progress of high magnetic field generation techniques and their use in industrial, medical, and scientific applications, the need for reliable miniaturized sensors that operate over a wide range of magnetic fields and are not sensitive to temperature variations has increased. The figure of merit of high-field sensors is the magnetoresistance value at different temperatures and magnetic fields and its linearity with the field. Therefore, the saturation of the *MR* at low and intermediate magnetic fields, which is typical for materials with a perfect crystalline structure, limits their application in high-field ranges. It was demonstrated that the introduction of some disorder in the form of defects, impurities or grain boundaries could be a way to extend the *MR* saturation limit and the high-field operation range of magnetic sensors.

**Figure 21:**
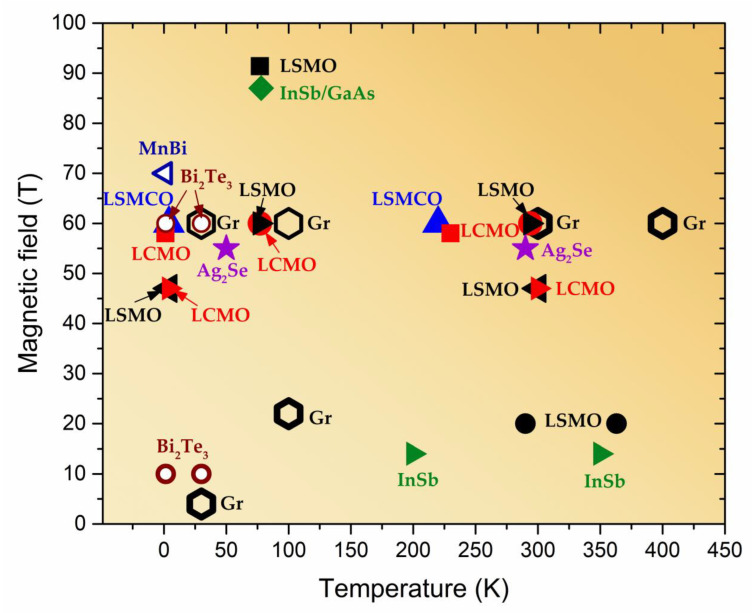
Overview of magnetic field and temperature ranges in which some classes of magnetic and non-magnetic magnetoresistive materials (discussed in this review) could be used for the development of high magnetic field sensors. Data are taken from different literature sources indicating magnetic fields and temperatures at which the magnetoresistance measurements were performed. LSMO, LCMO and LSMCO stand for manganites La-Sr-Mn-O and La-Ca-Mn-O and La-Sr-Mn-Co-O, respectively, Gr—graphene. The same symbol, but with thinner borders as shown for graphene and Bi_2_Te_3_, indicate that quantum oscillations superimposed on *MR*(*B*) dependence were observed in high fields at low temperatures. The symbols correspond to the following references for LSMO: 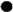 [11], 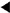 [54], 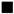 [62], 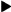 [75]; for LCMO: 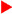 [72], 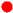 [75], 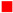 [76]; for LSMCO: 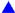 [78]; for InSb 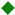 [21], 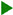 [111]; for Ag_2_Se 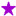 [104]; for MnBi 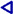 [126]; for Bi_2_Te_3_ 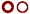 [127]; for Gr 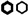 [128].

Figure 21 presents a range of magnetic field in which magnetoresistance measurements were performed for typical material classes discussed in this review. For more details see Table 1 and Table 2. The *X*-axis of the graph presents the temperature or its range (the same symbols represent the range borders) at which the measurements were performed. The materials are indicated close to the corresponding symbols.

It is shown, that colossal magnetoresistive materials (manganites) can be used up to megagauss field in a wide range of temperatures. For fabrication of high-field sensors, the polycrystalline or nanostructured films are required. The formation of structurally and magnetically disordered grain boundaries leads to not fully saturating, albeit non-linear, *MR* dependence up to megagauss. The maximum temperature of operation of CMR-sensors is approximately (360–370) K for nonstoichiometric films with Mn excess. The main advantages of the CMR-sensors are small dimensions and isotropic behavior: the so-called CMR-B-scalar sensors are capable of measuring magnetic field magnitude independently on the field orientation with respect to the sensor’s plane.

Another group of materials are silver chalcogenides in which the introduction of a small amount of excess silver (approximately one part in 100,000) leads to extremely large, linear magnetoresistance that does not saturate up to megagauss. The magnetic sensors based on silver chalcogenides could be used up to room temperatures. However, at *T* < (30–50) K, the quantum oscillations emerge on the *MR* dependence which limit the accuracy of these sensors and possibilities to use these devices at cryogenic temperatures.

Magnetic sensors based on narrow bandgap semiconductors, such as InSb, with introduced inhomogeneities also exhibit linear magnetoresistance up to high fields; however, due to quantum effects at low temperatures, the operation temperature range of (200–350) K is preferable.

In recent years, the most attention was drawn to 2D materials, such as graphene and transition metal dichalcogenides, due to the possibility of scaling down the sensing area of the devices to micrometer or nanometer dimensions. It was demonstrated that the small amount of disorder can result in linear magnetoresistance in bilayer graphene up to 62 T at temperatures up to 400 K. Moreover, the *MR* of graphene is not very sensitive to temperature variations. However, at cryogenic temperatures, the quantum oscillations limit the performance of graphene-based sensors.

The few data in the Figure 21 are shown for a special class of materials—topological insulators (Bi_2_Te3) and ferromagnetic semimetals (MnBi). Recently, the research of their fundamental properties and novel phenomena has received a lot of attention, however, their application in magnetic sensing is limited due to significant *MR* vales only at very low temperatures. On the other hand, for other spintronics applications these materials are promising due the richness of their unusual properties.

The possibility to fabricate the magnetoresistive sensors with two-terminal design is an advantage for pulsed-field measurements avoiding significant pick-up noise induced by large d*B*/d*t* values due to high-amplitude and short duration magnetic field pulses. Pick-up coils require signal integration which limits the sensor’s accuracy, while magnetoresistive sensors provide absolute calibration. Moreover, with the increasing need of sensor miniaturization for local measurements in various medical and industrial equipment, the relatively large pick-up coils are not applicable for opening the path for solid-state materials-based sensors.

In conclusion, the engineering of advanced high carrier mobility materials on microscales by exploiting the effects of disorder has the potential for the development of future magnetic field sensors with extended application ranges to strong and ultra-strong magnetic fields (1–100 T) and high temperatures, at which the phonon scattering is a limiting factor in usual devices based on perfect structure materials. Reliable control of the level of disorder ensures development of easy scalable magnetic field sensors. Comprehensive future investigations are needed for advanced 2D magnetoresistive materials and structures to optimize and stabilize their parameters over the time. Cheaper fabrication technologies must be developed which can be scaled from the laboratory to the industrial level.

## Figures and Tables

**Table 1:** Overview of high magnetoresistance magnitudes in polycrystalline manganites measured at low and close to room temperatures in high magnetic fields. The *MR* at intermediate temperature corresponding to the highest *MR* value is marked by *.

Composition	Preparation Peculiarities	Temperature, K	Magnetic Field, T	Magnetoresistance Magnitude, %	Reference
La_0.7_Ca_0.3_MnO_3_	Nano-crystalline	4–150	15–47	80−98	[72]
		* 150	47	~98	
		302	47	~68	
	Micro-crystalline	4.2	47	~80	
		* 250	47	~92	
		300	47	~85	
La_0.59_Ca_0.41_MnO_3_	Film; Substrate: lucalox (glass ceramics)	77	60	~95	[75]
		294	60	~80	
La_0.72_Ca_0.28_Mn_0.98_O_3_	Film; Substrate: lucalox; gas pressure in growth chamber 3 Torr	1.3	60	85	[76]
		* 225	60	98	
		290	60	85	
	Film; Substrate: lucalox; gas pressure in growth chamber 7 Torr	1.3	60	75	
		* 225	60	92	
		290	60	80	
La_2/3_Sr_1/3_MnO_3_	Ceramics, average size 25 nm	10	5.5	50	[70]
La_0.8_Sr_0.2_MnO_3_	Nano-crystalline	4	47	85	[54]
		* 157	47	89	
		300	47	70	
	Micro-crystalline	4.2	47	71	
		* 260	47	83	
		300	47	78	
La_0.83_Sr_0.17_MnO_3_	Film; Substrate: glass ceramics; film thickness 25 nm	77	20	81	[56]
		* 130	20	87	
		294	20	31	
	Film; Substrate: glass ceramics; film thickness 400 nm	77	20	53	
		* 230	20	71	
		294	20	62	
La_0.83_Sr_0.17_MnO_3_	Film; Substrate: lucalox (glass ceramics); film thickness 400 nm	77	91.4	78	[62]
		290	58	85	
La_0.82_Sr_0.18_Mn_1.15_O_3_	Film; Substrate: polycrystalline Al_2_O_3_; film thickness 360 nm; Mn excess 1.21	77	20	56	[68]
		* 250	20	66	
		363	20	38	
La_0.79_Sr_0.21_Mn_1.05_Co_0.12_O_3_	Film; Substrate: polycrystalline Al_2_O_3_; Co/(La + Sr) = 0.12	4	60	82	[78]
		* 220	60	83	
La_0.67_Ba_0.33_MnO_3_	ceramics	4.2	8	50	[80]
		294	8	29	
La_0.4_Gd_0.1_Ca_0.5_MnO_3_	ceramics	5	10	60–85	[81]
		125	10	98	
La_0.45_Ho_0.05_Ca_0.5_MnO_3_	ceramics	130	8	18	[82]
La_0.45_Ca_0.55_MnO_3_	Nanoparticles; average size 70 nm	80	7	99%	[83]
La_0.5_Ca_0.4_Li_0.1_MnO_3_	Grain size 5–10 μm	5	14	82	[84]
		300	14	30	

**Table 2:** Some examples of magnetoresistance magnitude in single, few-layer and multi-layered graphene measured at low and room temperatures in high magnetic fields.

Number of Graphene Layers	Substrate; Preparation Peculiarities	Temperature, K	Magnetic Field, T	Magnetoresistance Magnitude, %	Reference
1	SiO_2_; exfoliation from Kish graphite	300	9	200	[123]
4	SiO_2_, wet transfer	300	6	27	[129]
	SiO_2_, wet transfer; surface decoration with Co particles			36	
4	SiO_2_; mechanically peeling Kish graphite and transfer on SiO_2_	400	9	330	[132]
1	SiC; growth by thermal sublimation of substrate; change of annealing time	300	9	40–80	[122]
	SiC; growth by thermal sublimation of substrate	2		100	
multilayer	Grown on SiC	300	9	80	[120]
		4.2	12	250	
2	SiC; hydrogen intercalation	300	62	90	[128]
1	Polycrystalline Al_2_O_3_; wet transfer	300	9	160	[12]
			20	450	
3			9	325	
			20	760	
1	Black phosphorus (BP); dry transfer	300	9	780	[123]
4	Boron nitride (BN); mechanical peeling of Kish graphite and transfer on BN	400	10	900	[132]
6				2000	
		2	12	6000	
1	SrTiO_3_; laminating on terraced substrate	300	9	5000	[124]

## Data Availability

No new data were created as it is a review paper.

## Abbreviations

Acronym/abbreviation	Definition
1LG	Single layer graphene
2D	Two-dimensional
3LG	Three-layer graphene
AMR	Anisotropic magnetoresistance
BP	Black phosphorus
CMR	Colossal magnetoresistance
EMR	Extraordinary magnetoresistance
FET	Field-effect transistor
GMI	Giant magnetoimpedance
GMR	Giant magnetoresistance
Gr	Graphene
h-BN	hexagonal boron nitride
HFMR	High-field magnetoresistance
LCMO	La_1−x_Ca_x_MnO_3_
LFMR	Low-field magnetoresistance
LMR	Linear magnetoresistance
LSMO	La_1−x_Sr_x_Mn_y_O_3_
LSMCO	La_1−x_Sr_x_(Mn_1−y_Co_y_)_z_O_3_
MRA	Magnetoresistance anisotropy
MTJ	Magnetic tunnel junction
MR	Magnetoresistance
SdHO	Shubnikov-de Haas oscillations
SEM	Scanning electron microscopy
PL	Parish and Littlewood model
TEM	Transmission electron microscopy
TMDC	Transition metal dichalcogenide
TMR	Tunneling magnetoresistance
TRL	Technology readiness levels
VRH	Variable range hopping
